# Spatiotemporal drivers of Nature's contributions to people: A county-level study

**DOI:** 10.1016/j.ese.2024.100430

**Published:** 2024-05-19

**Authors:** Wei Jiang, Bojie Fu, Zhongguo Shu, Yihe Lv, Guangyao Gao, Xiaoming Feng, Stefan Schüler, Xing Wu, Cong Wang

**Affiliations:** a State Key Laboratory of Urban and Regional Ecology, Research Center for Eco-Environmental Sciences, Chinese Academy of Sciences, No.18 Shuangqing Road, 100085, Beijing, China; b Functional Agrobiodiversity, Georg-August-Universität Göttingen, Grisebachstraße 6, 37077, Göttingen, Germany

**Keywords:** Potential regulating contributions, Natural and anthropogenic drivers, Path analysis, Trade-off or synergy relationship, China

## Abstract

Nature's contributions to people (NCP) encompass both the beneficial and detrimental effects of living nature on human quality of life, including regulatory, material, and non-material contributions. Globally, vital NCPs have been deteriorating, accelerated by changes in both natural and anthropogenic drivers over recent decades. Despite the often inevitable trade-offs between NCPs due to their spatially and temporally uneven distributions, few studies have quantitatively assessed the impacts of different drivers on the spatial and temporal changes in multiple NCPs and their interrelationships. Here we evaluate the effects of precipitation, temperature, population, gross domestic product, vegetation restoration, and urban expansion on four key regulatory NCPs—habitat maintenance, climate regulation, water quantity regulation, and soil protection—in Nei Mongol at the county level. We observe increasing trends in climate regulation and soil protection from 2000 to 2019, contrasted with declining trends in habitat maintenance and water quantity regulation. We have identified the dominant positive and negative drivers influencing each NCP across individual counties, finding that natural drivers predominantly overpowered anthropogenic drivers. Furthermore, we discover significant spatial disparities in the trade-off or synergy relationships between NCPs across the counties. Our findings illustrate how the impacts of various drivers on NCPs and their interrelationships can be quantitatively evaluated, offering significant potential for application in various spatial scales. With an understanding of trade-offs and scale effects, these insights are expected to support and inform policymaking at both county and provincial levels.

## Highlights

• NCP4 and NCP8 show increasing trends whereas NCP1 and NCP6 declining trends.
• NCP and drivers exhibit spatial heterogeneity and temporal variability.
• Precipitation is the most significant driver, followed by temperature.
• Dominant positive and negative drivers of NCP are identified.
• Trade-off or synergy relationships between NCP are identified.

## Graphical abstract

**Figure:**
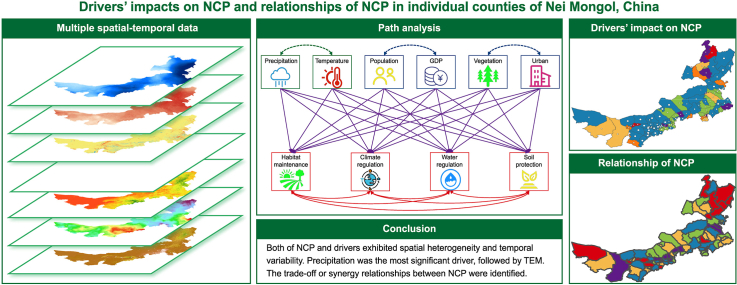

## 1 Introduction

Building upon the concept of ecosystem service (ES) established by Costanza et al. [1] and popularized by the Millennium Ecosystem Assessment [2], the concept of nature's contributions to people (NCP) was put forward by the Intergovernmental Science-Policy Platform on Biodiversity and Ecosystem Services (IPBES) as a fundamental element of the conceptual framework [3]. NCP is defined as all the beneficial and detrimental contributions of living nature to people's quality of life. Eighteen categories of NCP have been identified and classified into three partially overlapping groups: material, non-material, and regulating contributions [4,5]. NCP is further distinguished between potential NCP, which refers to the capacity of ecosystems to provide NCP, and realized NCP, which refers to the actual flow of NCP people obtain [6,7]. This distinction depends on people's needs, suggesting that the overlap between potential NCP and people's needs corresponds to realized NCP [8].

From the methodological perspective, the major difference between the assessment of NCP and ES is using indicators to represent NCP instead of using models (e.g., InVEST, SWAT, and ARIES) for calculating ES [9]. An indicator is synthetic information that qualitatively or quantitatively represents an object's status, process, cause, or outcome. Depending on the complexity of the object and the level of aggregation, indicators range from directly measurable parameters to comprehensive indices [10]. Using standardized indicators for assessing NCP is helpful for monitoring, comparing, and communicating changes in different aspects of nature over time [6]. The indicator approach with a multi-dimensional system of indicators developed by the IPBES has been adopted in recent studies [7,11,12].

Research on the impact of multiple driving factors on ES changes has recently drawn increasing attention. For example, Ouyang et al. [13] found that economic investments in protecting and restoring ecosystems realized considerable ES improvements in China, Serna-Chavez et al. [14] identified climate, soil, forest fragmentation, and land use change as the major drivers of climate regulation in the Americas. Wilkerson et al. [15] showed that native culture, government policies, and technological progress strongly affected spatial distributions of ES supply and demand. Meanwhile, multiple driving factors often exert a cumulative and simultaneous effect. For example, economic, social, environmental, technological, and governance factors played a cumulative role in ES trade-offs in European wood pastures [16], and spatial-temporal differentiation of topographic, climatic, and socio-economic factors influenced ES distribution in the Pearl River Delta of China [17].

The IPBES global assessment showed that vital NCP are deteriorating worldwide. Most material NCP has increased over the past 50 years, while most regulating and non-material NCP have decreased [6]. Multiple natural and anthropogenic drivers have accelerated NCP changes since 1970, such as land use change, climate change, population growth, and economic development. Furthermore, trade-offs among NCP are often inevitable in different regions and segments of society because of the uneven distribution of NCP across time and space [6]. The release of this global assessment has drawn wide attention to the research on NCP. For example, Des Roches et al. [18] suggested that the main categories of NCP are supported by intraspecific variation. O'Connor et al. [19] identified the potential priority areas for conserving biodiversity, improving culture, and regulating NCP in Europe. Cimatti et al. [12] showed that the global high-biodiversity regions would be of great importance for providing air quality regulation, climate regulation, and freshwater quantity regulation under future climate change. However, these studies were mainly focused on revealing the effect of biodiversity on providing NCP. In contrast, there have still been few studies that quantitatively estimate the impact of different drivers on the spatial and temporal changes in NCP and identify the varying relationships of multiple NCP in different regions.

Therefore, this study attempts to fulfill this research gap and expand the research subjects of NCP. The purposes of this study include (1) mapping spatial-temporal changes in potential NCP and their major drivers, (2) estimating the impact of natural and anthropogenic drivers on the changing potential NCP, and (3) quantifying the relationships between these potential NCP. We take Nei Mongol Zizhiqu (Nei Mongol) in China as a case study because it serves as an important ecological shelter in northern China's arid and semi-arid regions [20]. The natural environment in Nei Mongol is sensitive and vulnerable to global climate change due to scarce water resources and fragile habitats [21]. Combined with increasing human activities, this agro-pastoral transition zone has long suffered from severe land degradation and frequent sand storms [22], significantly influencing regional food production and socio-economic development [23] and seriously limited residents' livelihood improvement [24]. Large-scale ecological restoration programs in China have been implemented to combat desertification, improve environmental quality, and promote economic development in Nei Mongol [25].

## 2 Materials and methods

### 2.1 Study area

**Fig. 1:**
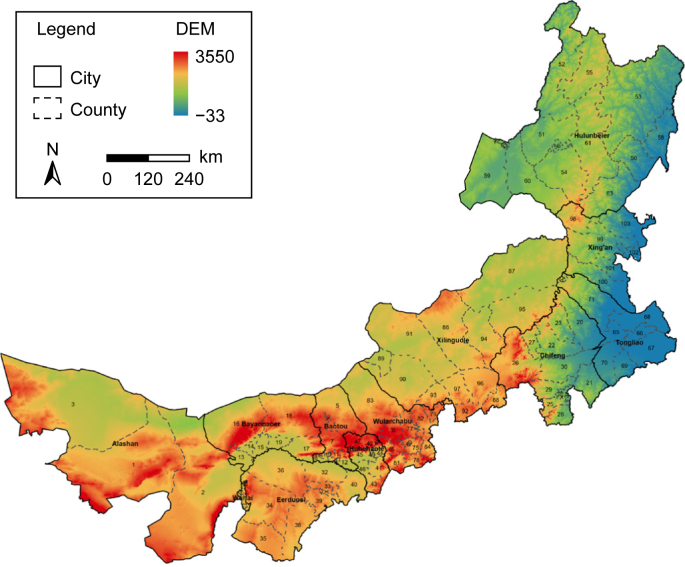
Geography of Nei Mongol Zizhiqu.

Nei Mongol is the third largest province of China, spanning in the northern China (97°–126° E and 37°–53° N) and covering ca. 1.18 million km^2^. With an average elevation of over 1000 m, the topography shows a visible pattern of high in the west and low in the east. Nei Mongol contains 103 banners and counties within 12 prefecture-level leagues and cities (Fig. 1). Banners and leagues are specific administrative units in Nei Mongol, corresponding to counties and prefecture-level cities in other provinces. The counties' information, including their names, areas, abbreviations, and Chinese names, can be found in Table S1. Located within the temperate continental monsoon climatic zone, the climate in Nei Mongol varies from arid and semi-arid in the west to semi-humid in the east, with an annual mean precipitation of 50–550 mm that increases from southwest to northeast and an annual mean temperature of 3–6 °C that declines from south to north [22].

### 2.2 Analysis framework

**Fig. 2:**
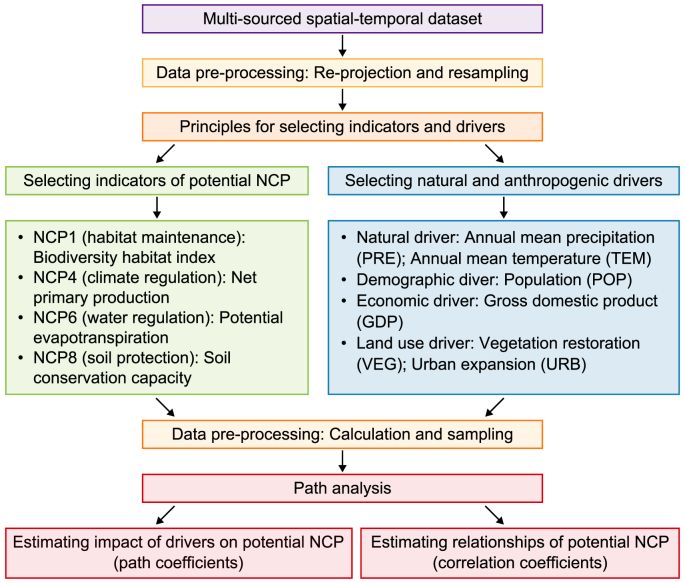
A general framework for conducting path analysis.

We develop a general research framework for path analysis (Fig. 2). This framework starts by collecting multi-sourced spatial-temporal datasets and preprocessing them. By adopting selection principles, indicators of potential NCP and natural and anthropogenic drivers are selected. After data calculation and sampling, they are used in the path analysis.

#### 2.2.1 Data collection and pre-processing

The spatial-temporal datasets from multiple sources are summarized in Table 1. To make them consistent and align each other, ArcGIS Pro 3.0.1 was used to perform necessary pre-processing, including transferring all raster datasets to the same geographic and projected coordinate system and resampling them to the same spatial resolution of 1000 m.

**Table 1:** Multi-sourced spatial-temporal datasets.

Dataset (unit)	Spatial resolution	Temporal resolution	Reference
Biodiversity habitat index (score)	1000 m	2000, 2020^a^	[26]
Net primary productivity (gC m^−2^ year^−1^)	500 m	2001^b^, 2019	[27]
Annual potential evapotranspiration (mm)	1000 m	2000, 2019	[28]
Soil conservation capacity (ton ha^−1^ year^−1^)	300 m	2000, 2019	[29]
Annual mean precipitation (mm)	1000 m	2000, 2019	[30]
Annual mean temperature (°C)	1000 m	2000, 2019	[31]
Population counts (number of people)	1000 m	2000, 2019	[32]
Gross domestic product (million US dollar of 2017^c^)	1000 m	2000, 2019	[33]
Land use (category)	30 m	2000, 2019	[34]

a The data for biodiversity habitat index in 2020 was adopted to replace that in 2019.

b The data for net primary productivity in 2001 was adopted to replace that in 2000.

c The unit was transferred to million yuan using the exchange rate of 6.7518 in 2017.

#### 2.2.2 Choosing indicators of potential NCP

We focused on changes in four main potential regulating contributions from 2000 to 2019: habitat maintenance (NCP1), climate regulation (NCP4), water quantity regulation (NCP6), and soil protection (NCP8), due to their importance Nei Mongol [20]. These contributions are globally facing declines [6]. Following the “4S2P” principles for choosing indicators — scientifically sound, sensitive to changes, suitable for (dis)aggregation, simply understandable, practically affordable, and policy-relevant [6] — we adopted the biodiversity habitat index (BHI), net primary production (NPP), potential evapotranspiration (PET), and soil conservation capacity (SCC) to indicate NCP1, NCP4, NCP6, and NCP8, respectively.

BHI estimates the expected persistence of species diversity within a given spatial unit based on the variations in species composition and the changes in ecosystem integrity and connectivity, representing the effective proportion of remaining habitat within this spatial unit. By monitoring temporal trends in the expected level of retained species diversity in different years, BHI is useful for assessing the effect of ecological protection actions planned or implemented to enhance ecosystems' ability to maintain biodiversity [26]. A larger BHI indicates a higher provision of NCP1.

NPP is defined as the difference between the amount of carbon assimilated by photosynthesis and the amount of carbon released by plant respiration, measuring the level of CO_2_ accumulation from the atmosphere to terrestrial ecosystems [35] and the increments in biomass per unit of land and time [12]. NPP is an important component of carbon balance, thus serving as an appropriate indicator of ecosystem climate regulation. The larger NPP means the higher provision of NCP4.

PET is defined as the amount of water that can be transferred from the land surface to the atmosphere through two simultaneous processes of evaporation and transpiration without limiting available water [36,37]. PET measures the capacity of the atmosphere to remove water from land, thus serving as an indicator of water quantity regulation but in a reverse direction [12]. A larger value of PET represents a lower provision of NCP6.

SCC measures the difference between the amount of potential and actual soil erosion based on the Revised Universal Soil Loss Equation, representing the ability of ecosystems to conserve soils by vegetation management and support practice [29]. SCC is a critical indicator for protecting multiple ecological dimensions supported by ecosystems [38]. A larger value of SCC is associated with a higher provision of NCP8.

#### 2.2.3 Selecting natural and anthropogenic drivers

Drivers refer to all direct or indirect factors that positively or negatively affect the provision of potential NCP, and they can be classified into natural and anthropogenic drivers [6]. The principles for choosing indicators of potential NCP can also be adapted to select relevant drivers. Instead of being sensitive to changes, drivers should inherently vary both spatially and temporally. This approach excludes factors typically considered static, such as elevation and slope. With the suitable for (dis)aggregation, drivers should also be mapped at the grid level in line with gridded potential NCP. Hence, it is possible to quantitatively determine the impact of temporal variations in drivers during a given period on temporal changes in NCP during the same period [39]. Based on these considerations, we selected six drivers, including two natural drivers (annual mean precipitation [PRE] and annual mean temperature [TEM]), one demographic driver (population [POP]), one economic driver (gross domestic product [GDP]), and two land use drivers (vegetation restoration [VEG] and urban expansion [URB]). Table 2 summarizes the abbreviations used for indicators of potential NCP and drivers.

**Table 2:** Abbreviations used for indicators and drivers.

Type of potential NCP	Indicator	Abbreviation
NCP1 habitat maintenance	Biodiversity habitat index	BHI
NCP4 climate regulation	Net primary production	NPP
NCP6 water quantity regulation	Potential evapotranspiration	PET
NCP8 soil protection	Soil conservation capacity	SCC
Type of driver	Driver	Abbreviation
Natural driver	Annual mean precipitation	PRE
	Annual mean temperature	TEM
Demographic driver	Population	POP
Economic driver	Gross domestic product	GDP
Land use driver	Vegetation restoration	VEG
	Urban expansion	URB

#### 2.2.4 Data calculation and sampling

The land use dataset with 30 m spatial resolution was used to calculate VEG and URB by ArcGIS Pro 3.0.1. First, we created a fishnet with 1000 × 1000 m and kept it consistent with other raster datasets. Second, we extracted the land use categories of forest, shrub, grassland, and built-up land from the land use datasets. Third, we calculated the areas of these land use categories in each fishnet grid using zonal statistics. Finally, the ratio between the area of forest, shrub, grassland, and the area of a grid (10^6^ m^2^) was calculated to represent VEG, while the ratio between the area of built-up land and the grid area was calculated to represent URB. The spatial distribution of land use in Nei Mongol in 2000 and 2019 is shown in Fig. S1.

Due to limitations in hardware and software capacity, data sampling was necessary for the path analysis. A total of 1155983 grids, each with a spatial resolution of 1000 m, cover the whole Nei Mongol. Each grid serves as a sample, containing ten variables (i.e., four indicators of potential NCP and six drivers). We used the random sampling technique in ArcGIS Pro 3.0.1 to choose 1000 samples in each county. Using the “extract multi values to points” in ArcGIS Pro 3.0.1 and excluding the samples with null values, the sample size amounted to 86621 across Nei Mongol, accounting for ca. 7.5% of the total grids. Several counties with small areas contained less than 1000 grids. For example, the smallest county, Jining, contained 52 samples; Wuda contained 99 samples, and Yuquan contained 104 samples. The sample size for each county in Nei Mongol is detailed in Table S1.

#### 2.2.5 Path analysis

Ecologists usually attempt to clarify relationships among variables that implicitly underlie complex and unclear interactions. The structural equation model is an ideal approach for this task because it aims at generating a single network to explore the interactions of multiple variables [40]. The structural equation model family includes specific methods with differences among their relative focus on observed and unobserved variables, longitudinal and hierarchical data, and fitting approaches [41]. Focused solely on observed variables, path analysis can quantify the relationships among a set of variables based on a particular working model about their interactions. It has been proved as an accessible and feasible technique used by ecologists [39,42].

**Fig. 3:**
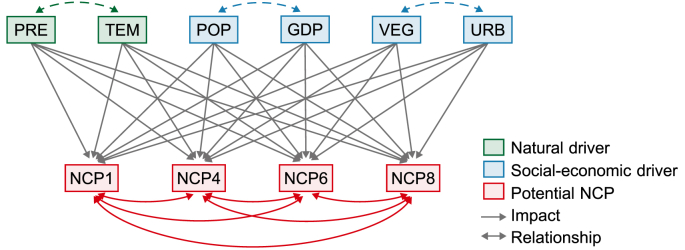
Path diagram for impacts of drivers on potential NCP and relationships between potential NCP.

By constructing an a priori path diagram (Fig. 3), the impact of a driver on a potential NCP is reflected by a straight single-headed arrow (e.g., from VEG to NCP1) with a path coefficient that quantitatively indicates the degree of influence, while the relationship between two potential NCP is reflected by a curved double-headed arrow (e.g., between NCP2 and NCP4) with a correlation coefficient that quantitatively indicates the level of relationship [43]. Both path and correlation coefficients can be positive and negative: a positive/negative path coefficient denotes the positive/negative impact of a driver on a potential NCP, while a positive/negative correlation coefficient denotes the synergy/trade-off relationship between two potential NCP. The widely used maximum likelihood method is adopted to estimate the parameters of the path analysis model because this estimation method can automatically be applied and generates optimal inference [44]. SPSS Statistics 27 and SPSS Amos 28 were used to implement the path analysis.

## 3 Results

### 3.1 Spatial-temporal changes in potential NCP

From 2000 to 2019, an increasing trend was found in NCP4 and NCP8, while a slight decreasing trend was found in NCP1 and NCP6 in Nei Mongol. The mean BHI decreased by 0.01. The mean PET showed an increase of 3.99 mm, representing a decrease of NCP6. In contrast, the mean NPP increased by 74.98 gC m^−2^ year^−1^, and the mean SCC showed a rise of 32.47 ton ha^−1^ year^−1^ (Table 3).

**Table 3:** Changes in potential NCP in Nei Mongol from 2000 to 2019.

Indicator (unit)	2000	2019	Change between 2000 and 2019	Potential NCP
BHI (score)	0.87	0.86	−0.01	NCP1 decrease
NPP (gC m^−2^ year^−1^)	150.34	225.32	74.98	NCP4 increase
PET (mm)	937.48	941.47	3.99	NCP6 decrease
SCC (ton ha^−1^ year^−1^)	19.46	51.93	32.47	NCP8 increase

**Fig. 4:**
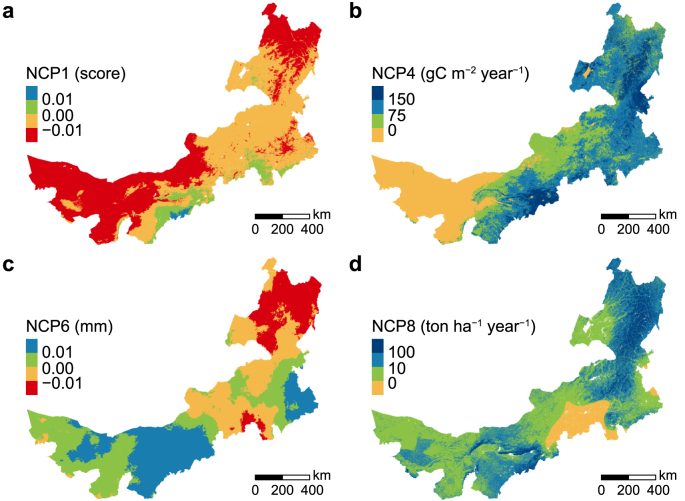
Spatial changes in levels of four potential NCP at 1000 m resolution from 2000 to 2019. **a**, NCP1 habitat maintenance (score); **b**, NCP4 climate regulation (gC m^−2^ year^−1^); **c**, NCP6 water quantity regulation (mm); **d**, NCP8 soil protection (ton ha^−1^ year^−1^). Red and orange represent negative changes, and green and blue represent positive changes.

These overall changing trends in the whole Nei Mongol conceal high spatial heterogeneity in the distribution of potential NCP. The significant decrease of NCP1 (≤−0.01) was found in the northeast and central west of the Nei Mongol, while the increase was found in the central south and southeast (Fig. 4a). NCP4 decreased (≤0 gC m^−2^ year^−1^) in the western region and significantly increased (>150 gC m^−2^ year^−1^) in the northeast and central south (Fig. 4b). NCP6 showed considerable declines (>15 mm) in the eastern, central, and western regions and large increases (≤−15 mm) mainly in the northeast and southeast (Fig. 4c). The decrease of NCP8 (≤0 ton ha^−1^ year^−1^) was mainly seen in the central south, while the largest increase (>100 ton ha^−1^ year^−1^) was in the northeastern and central regions (Fig. 4d). The spatial distribution of four potential NCP in Nei Mongol in 2000 and 2019 were shown in Fig. S2.

### 3.2 Spatial-temporal variations in drivers

From the statistical perspective of the whole Nei Mongol, all six drivers showed increasing trends from 2000 to 2019. The annual mean precipitation increased by 69.63 mm, and the annual mean temperature increased by 1.12 °C. The total population experienced a slight increase of 0.43 million persons, while the total gross domestic product saw a considerable growth of 1567.34 billion yuan. The total vegetation area (including forest, shrub, and grassland) and total urban area exhibited an increase of 7372.56 and 4985.37 km^2^, respectively (Table 4).

**Table 4:** Variations of drivers in Nei Mongol from 2000 to 2019.

Driver (unit)	2000	2019	Change between 2000 and 2019
Annual mean precipitation (mm)	234.21	303.84	69.63
Annual mean temperature (°C)	3.96	5.08	1.12
Total population (million persons)	23.72	24.15	0.43
Total gross domestic product (billion yuan)	153.91	1721.25	1567.34
Total vegetation area (km^2^)	724736.90	732109.46	7372.56
Total urban area (km^2^)	5371.50	10356.86	4985.37

**Fig. 5:**
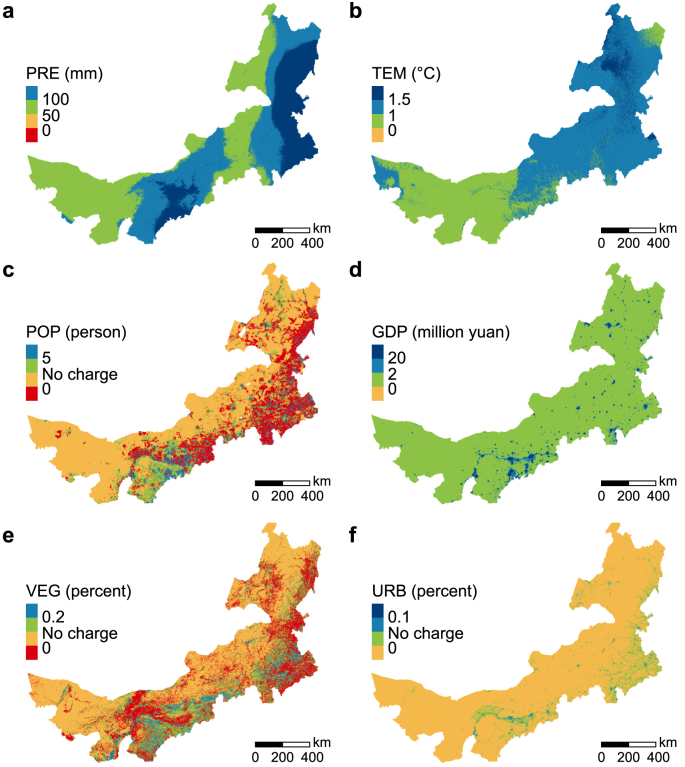
Spatial variations in levels of six drivers at 1000 m resolution from 2000 to 2019. **a**, PRE (annual mean precipitation, mm); **b**, TEM (annual mean temperature, °C); **c**, POP (population, person); **d**, GDP (gross domestic product, million yuan); **e**, VEG (vegetation restoration, %); **f**, URB (urban expansion, %). Red represents negative changes, orange represents no change, and green and blue represent positive changes.

From the spatial perspective of the grid level, the distribution of driver variations exhibited different patterns. The significant increase (>100 mm) of PRE was found in the northeast, southeast, and central south of the Nei Mongol (Fig. 5a), while the largest warming (>1.5 °C) occurred in the northeast (Fig. 5b). The change in POP showed high spatial heterogeneity, with declines (<0 person) in the southeastern and central southern regions and obvious increases (>5 person) mainly in the central south (Fig. 5c). Similar to POP, the change in VEG also showed a complex pattern, with reductions (<0%) in the northeastern, southeastern, and central-western regions and increases (>0.2%) in the southeast and central south (Fig. 5e). The growth of GDP (≤2 million yuan) prevailed in Nei Mongol with a sporadic distribution of significant growth (>20 million yuan; Fig. 5d), which highly corresponds with the spatial distribution of the considerable increase in URB (>0.1%; Fig. 5f). The spatial distribution of six drivers in Nei Mongol in 2000 and 2019 were shown in Fig. S3.

### 3.3 Absolute impact of drivers on potential NCP

**Fig. 6:**
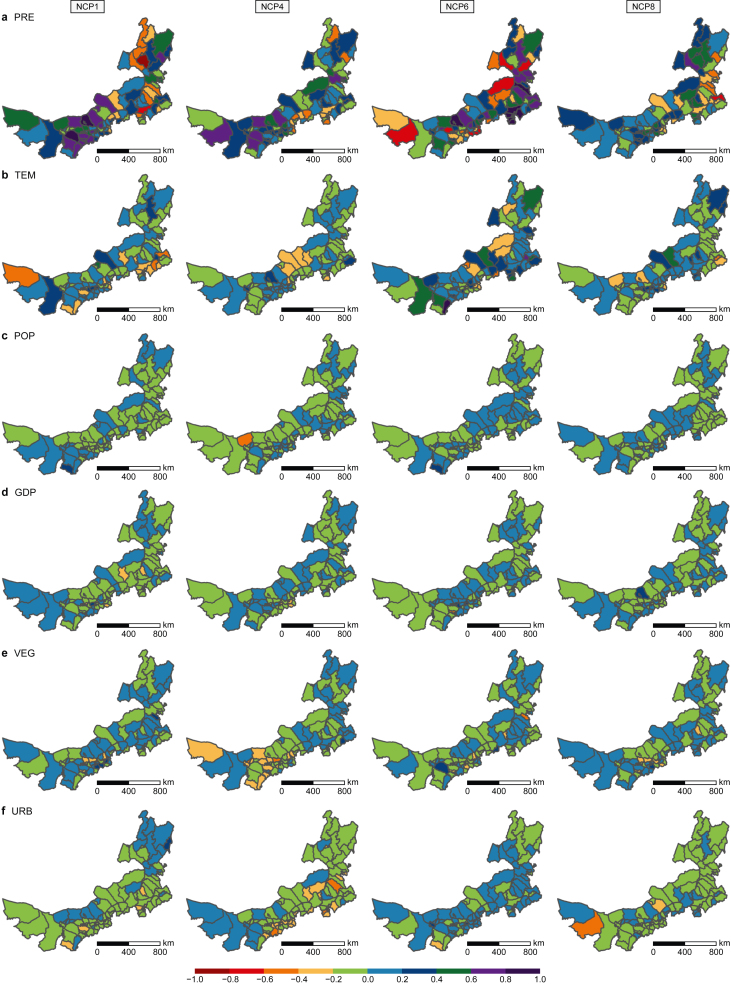
Absolute value of drivers' impact on potential NCP in each county. Impact of PRE (**a**), TEM (**b**), POP (**c**), GDP (**d**), VEG (**e**), and URB (**f**) on NCP1, NCP4, NCP6, and NCP8, respectively. PRE: annual mean precipitation; TEM: annual mean temperature; POP: population; GDP: gross domestic product; VEG: vegetation restoration; URB: urban expansion.

Fig. 6 illustrates the absolute impact of six drivers on four potential NCP in each county of Nei Mongol. PRE's impact on all four potential NCP was significant and highly varying (Fig. 6a). For example, the impact of PRE on NCP1 ranged from −0.83 in Ewenkezu (dark red in the first panel of Fig. 6a) to 0.90 in Daerhanmaominganlianhe and 0.85 in Hangjin (dark purple in the first panel of Fig. 6a). The impact of TEM on potential NCP was also relatively large (Fig. 6b). For example, the impact of TEM on NCP6 ranged from −0.55 in Erlianhaote and −0.43 in Duolun (orange in the third panel of Fig. 6b) to 0.98 in Wushen (dark purple in the third panel of Fig. 6b). Compared to the impact of two climate drivers, the drivers of human activities exerted moderate and steady influence on potential NCP, mostly ranging between −0.2 and 0.2 (dark green and yellow, respectively; Fig. 6c–f). Detailed information on the absolute impact value is shown in Table S2.

### 3.4 Relative importance of drivers on potential NCP

**Fig. 7:**
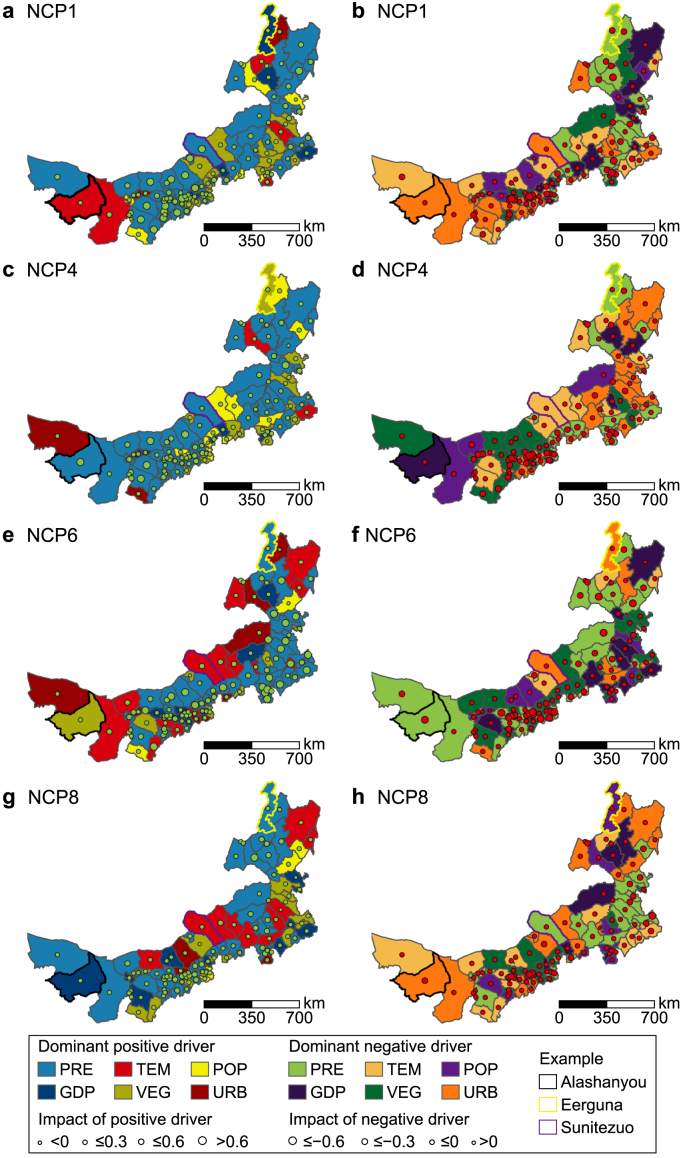
Dominant positive drivers and their degree of impact on NCP1 (**a**), NCP4 (**c**), NCP6 (**e**), and NCP8 (**g**), respectively. Dominant negative drivers and their degree of impact on NCP1 (**b**), NCP4 (**d**), NCP6 (**f**), and NCP8 (**h**), respectively.

It is more crucial to identify the relative importance of different drivers on potential NCP from the perspective of individual counties. We focused on the dominant positive drivers (i.e., drivers with the largest degree of impact) and dominant negative drivers (i.e., drivers with the smallest degree of impact) on four potential NCP. Fig. 7 illustrates both dominant positive and negative drivers on potential NCP and their degrees of impact in each county.

Considering spatial locations and illustrative visibility, we took three counties from the west to the east of Nei Mongol as examples: Alashanyou, Sunitezuo, and Eerguna. In Alashanyou, the dominant positive drivers on NCP1, NCP4, NCP6, and NCP8 were TEM (0.05), PRE (0.70), VEG (0.08), and GDP (0.05), respectively (Fig. 7 a, c, e, g), while the dominant negative drivers were URB (−0.05), GDP (−0.04), PRE (−0.68), and URB (−0.46), respectively (Fig. 7 b, d, f, h). The dominant positive drivers of Sunitezuo were PRE (0.77 on NCP1 and 0.30 on NCP4, respectively) and TEM (0.32 on NCP6 and 0.26 on NCP8, respectively) (Fig. 7 a, c, e, g), while the dominant negative drivers were URB on NCP1 (−0.09), TEM on NCP4 (−0.27), URB on NCP6 (−0.03), and PRE on NCP8 (−0.25) (Fig. 7 b, d, f, h). GDP, VEG, and PRE showed the dominantly positive impact on NCP1 (0.13), NCP4 (0.05), NCP6 (0.25), and NCP8 (0.25) in Eerguna (Fig. 7 a, c, e, g), while PRE, URB, and POP showed the dominantly negative impact on NCP1 (−0.46), NCP4 (−0.18), NCP6 (−0.09), and NCP8 (−0.16) (Fig. 7 b, d, f, h).

In addition, there were counties where the most significant driver had a negative impact on a given potential NCP; for instance, the largest impact of POP on HAB in Duolun was −0.052, implying that all drivers had a negative impact on this potential NCP. In contrast, some counties demonstrated that the least significant driver had a positive impact on a potential NCP; for example, the smallest impact of URB on SCC in Linxi was 0.024, implying that all drivers influenced this NCP positively. Detailed information on the dominant positive and negative drivers can be found in Table S3.

### 3.5 Quantitative relationships between potential NCP

**Fig. 8:**
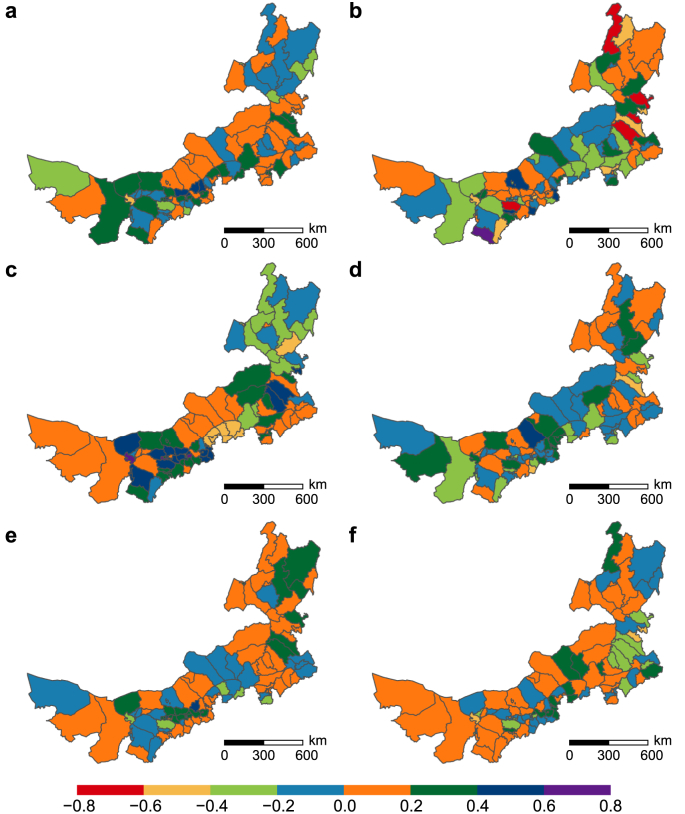
Quantitative pairwise relationships between potential NCP in each county. **a**, NCP1–NCP4; **b**, NCP1–NCP6; **c**, NCP1–NCP8; **d**, NCP4–NCP6; **e**, NCP4–NCP8; **f**, NCP6–NCP8.

Fig. 8 illustrates the pairwise relationships of four potential NCP in each county of Nei Mongol. The relationships between NCP1 and NCP4 was dominated by synergies, except for trade-offs distributing in the north-eastern and south-western counties (Fig. 7a). The relationships between NCP1 and NCP6 showed highly spatial heterogeneity, ranging from strong trade-offs (−0.8 to −0.6) in Eerguna, Dalate, Tuquan, Zhalute, and Zhalaite to strong synergies (0.6–0.8) in Etuokeqian (Fig. 7b). The trade-offs between NCP1 and NCP8 mainly distributed in the north-eastern and central-southern counties, while synergies prevailed in the most of counties (Fig. 7c). With moderate intensities of trade-offs and synergies, the spatial pattern of the relationships between NCP4 and NCP6 is similar to that between NCP1 and NCP6 (Fig. 7d). In contrast to the relationships between NCP1 and NCP8, the trade-offs between NCP4 and NCP8 were distributed in the central-western counties, while the synergies were distributed in the north-eastern and central-western counties (Fig. 7e). The counties in the north-east, south-east, and central showed trade-offs between NCP6 and NCP8, while other counties showed synergies (Fig. 7f). The detailed information on the value of pairwise relationships were shown in Table S4.

## 4 Discussion

### 4.1 Comparison to previous studies

This is the first study investigating the impact of different drivers on potential NCP and the relationships between potential NCP at the county level. Jiang et al. [39] explored the relationships between water yield and soil conservation in the West Liao River Basin, which is located in the southeast of Nei Mongol and covers 20 counties. They found that the intensities of synergy between water yield and soil conservation were 0.088, 0.026, and 0.074 in three sub-basins, respectively. Taking NCP6 as an equivalent to water yield and NCP8 as an equivalent to soil conservation, we showed the intensities of their relationships in each county in the West Liao River Basin. Among these intensities of relationships, the value in Wengniute (0.0874) is very close to that of the upstream of West Liao River Basin, and the value in Kailu (0.0295) is very close to that in the downstream of West Liao River Basin, while all the counties in the Wulijimuren River Basin showed trade-offs.

Dou et al. [20] estimated the relationship intensities between carbon sequestration, water retention, and soil retention in Nei Mongol at 0.31, 0.72, and 0.38, respectively. Considering NCP4, NCP6, and NCP8 as equivalents to these three ES, we can also quantify the relationships between NCP4, NCP6, and NCP8 for the whole region. The intensity between NCP4 and NCP6 was 0.17, approaching the value in Alukeerqin; the intensity between NCP4 and NCP8 was 0.21, approaching the value in Chaharrightfrontbanner; and the intensity between NCP6 and NCP8 was −0.03, approaching the value in Fengzhen (Table S4).

Compared to previous research, this study makes the following contributions (Table 5). First, the research object is shifted from ES to NCP (e.g., NCP4 vs. carbon sequestration, NCP6 vs. water yield, and NCP8 vs. soil conservation). Second, the assessment method is shifted from modeling (e.g., InVEST models) to indicators (e.g., IPBES indicator system). Third, establishing principles for selecting drivers provides a theoretical basis, suggesting that only varying drivers are considered to influence NCP changes. This allows all the drivers to be rasterized consistently with NCP, which are adopted as samples in path analysis. Finally, instead of commonly used tools for identifying the impact of drivers and the relationships between ES, including redundancy analysis [45], principal component analysis [46], geographical detector model [47], geographically weighted regression [48], and correlation analysis [49], path analysis is capable of integrally quantifying the impacts of drivers on NCP changes and the relationships between NCP.

**Table 5:** Contributions of this study in comparison to previous research.

Comparison item	Previous research	This study
Research object	ES	NCP
Assessment method	Modeling	Indicator
Principles for selecting drivers	No	Yes
Analyzing tool	Various statistical tools	Path analysis

On the other side, this study has several limitations. The primary limitation concerns data availability. In particular, spatialized socio-economic datasets are considerably less available than spatialized datasets of natural factors, constraining the selection of many other potential socio-economic drivers. The second issue involves the specification of the path analysis model, which is straightforward in this study without considering the possibility of instrumental variables that may help mitigate endogeneities and remove bias from estimates [50]. The third challenge is considering the first law of geography and spatial spillover effects, implying that regional attributes might be influenced by and simultaneously influence surrounding areas, especially in smaller administrative units such as counties [51]. The most important challenge is the scale-dependent effect, referring to the problem that the result of statistical analysis depends on adopted spatial units [52], such as geographic units (e.g., river basins or sub-basins) and administrative units (e.g., provinces or counties). Therefore, choosing appropriate temporal and spatial scales is important for the research objectives and analysis.

### 4.2 Implications for policymaking

Identifying key drivers on NCP changes and relationships enables decision-makers to consider adaptive ecosystem management strategies based on local conditions, aiming at either enhancing a certain NCP or optimizing the overall output of multiple NCP. Since multiple drivers often exert collective effects, the influence of different combinations of drivers on individual potential NCP is diverse. Taking the county of Sunitezuo as an example, the policymakers in this county are confronted with information about the quantitative impacts of six drivers on four potential NCP and quantitative relationships between four potential NCP. They would notice that the relationships of NCP1–NCP4, NCP1–NCP6, NCP1–NCP8, and NCP6–NCP8 are synergy, while relationships of NCP4–NCP6 and NCP4–NCP8 are trade-off (Fig. 7), suggesting that potential NCP4 should probably be a priority of improvement. They would then find out the respective impact of each driver on potential NCP4 from the largest to the smallest: PRE (0.30), POP (0.00), GDP (−0.02), VEG (−0.06), URB (−0.07), and TEM (−0.27) (Fig. 5, Table S2). PRE and TEM show the dominant positive and negative impact on potential NCP4, respectively, indicating that climate change plays the most significant role in providing NCP4. All GDP, VEG, and URB exhibit the constraining effect, implying that rapid economic development, vegetation restoration, and urban expansion may hamper the sustainable provision of NCP4. Policymakers should pay particular attention to vegetation restoration, assessing whether the chosen locations and structure of the vegetation are appropriate and can be further improved. Policymakers should consider the trade-offs and scale-dependent effects, striving to find strategies for achieving harmonious development between nature and society and balanced development across counties and provinces.

### 4.3 Perspectives on future work

First, a crucial advancement involves mapping realized NCP while considering human needs. Considering the spatial heterogeneity of human needs among counties, provinces, or countries, mapping realized NCP could better support policymaking at different local, regional, and national development strategies. Second, there is an urgent need for empirical research that quantifies the impact of nature on quality of life. Disentangling the influence of NCP on quality of life from various factors remains challenging. This requires information on the status and trends of the earth's system and how society interacts and coevolves with nature. Mapping the spatial distribution of benefits, which reflects divergent natural and socio-economic trends at local and regional scales, is particularly important. Finally, confronted with increasing risk induced by climate change, such as many recent extreme weather events, it is imperative to encourage a paradigm shift into a coupled social-ecological system, thus guiding the implementation of appropriate nature-based solutions for mitigating and adapting climate change.

## 5 Conclusions

This study is the first to quantify the impact of natural and anthropogenic drivers on multiple important potential nature's contributions to people (NCP) at the county scale. In Nei Mongol, our analysis revealed increasing trends for NCP4 and NCP8, alongside declining trends for NCP1 and NCP6, from 2000 to 2019. Concurrently, all six drivers exhibited increasing trends over the same period. Both the NCP and drivers exhibited spatial heterogeneity and temporal variability. Using the path analysis method, we estimated the impact of different drivers on four potential NCP, with precipitation (PRE) being the most significant driver, followed by temperature (TEM). Additionally, we identified the dominant positive and negative drivers of each potential NCP and identified the trade-off or synergy relationships between potential NCP in individual counties. With an awareness of trade-offs and scale effects, this information is helpful and supportive for policymaking at the county and provincial levels.

## CRediT authorship contribution statement

**Wei Jiang:** Conceptualization, Formal Analysis, Investigation, Methodology, Visualization, Writing - Original Draft. **Bojie Fu:** Supervision. **Zhongguo Shu:** Resources, Software. **Yihe Lv:** Supervision, Writing - Review & Editing. **Guangyao Gao:** Funding Acquisition, Project Administration. **Xiaoming Feng:** Funding Acquisition, Project Administration. **Stefan Schüler:** Writing - Review & Editing. **Xing Wu:** Writing - Review & Editing. **Cong Wang:** Writing - Review & Editing.

## Declaration of competing interest

The authors declare that they have no known competing financial interests or personal relationships that could have appeared to influence the work reported in this paper.
