# Supplementary material for: Spatiotemporal drivers of Nature's contributions to people: A county-level study
Source: Environ Sci Ecotechnol. 2024 May 19;20:100430. doi: 10.1016/j.ese.2024.100430 (PMC11153088; doi:10.1016/j.ese.2024.100430)
Supplement: Multimedia component 1 [file mmc1.docx]

Figure S1. The spatial distribution of land cover types in Nei Mongol in 2000 and 2019.


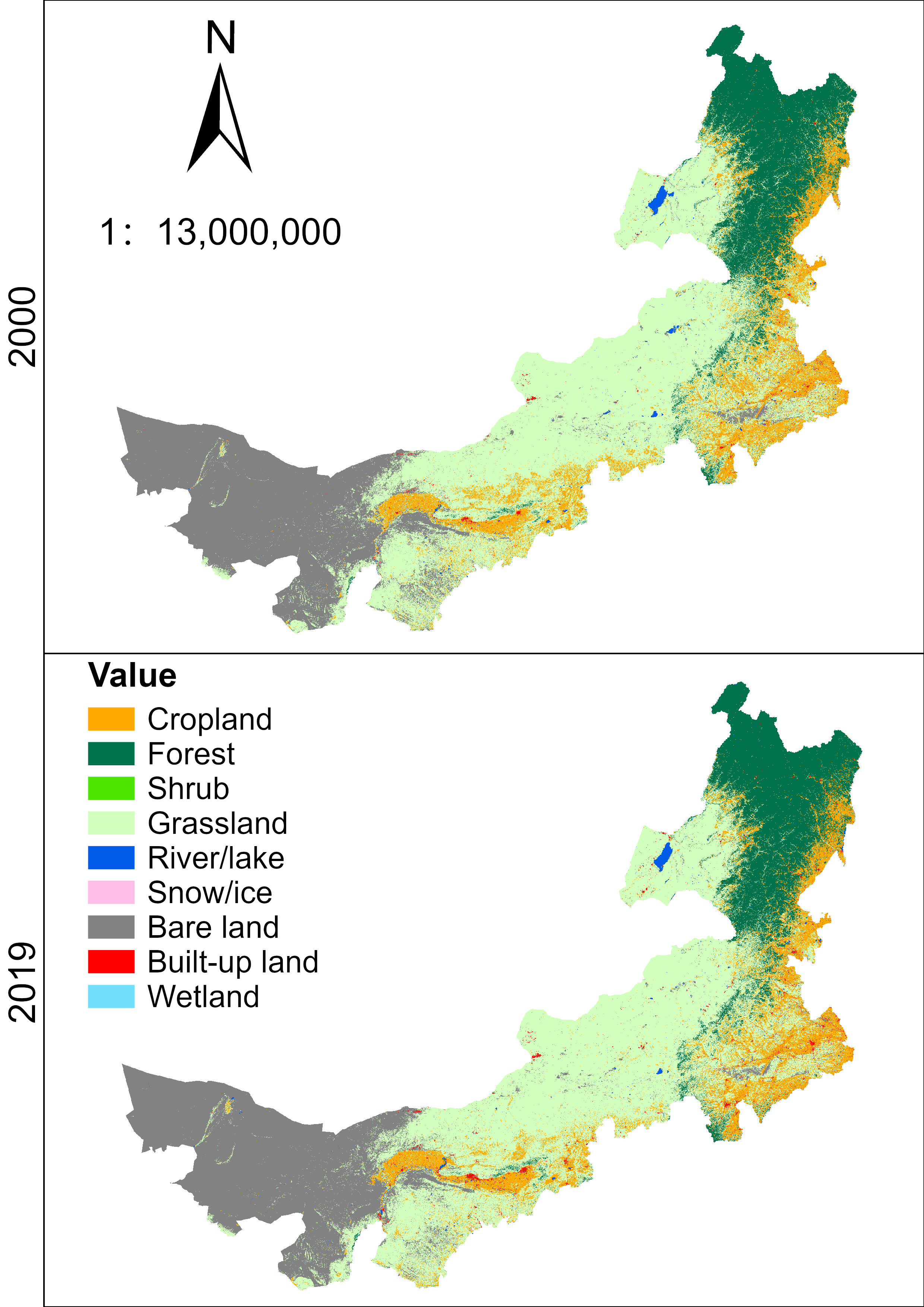


Figure S2. The spatial distribution of four potential NCP in Nei Mongol in 2000 and 2019.


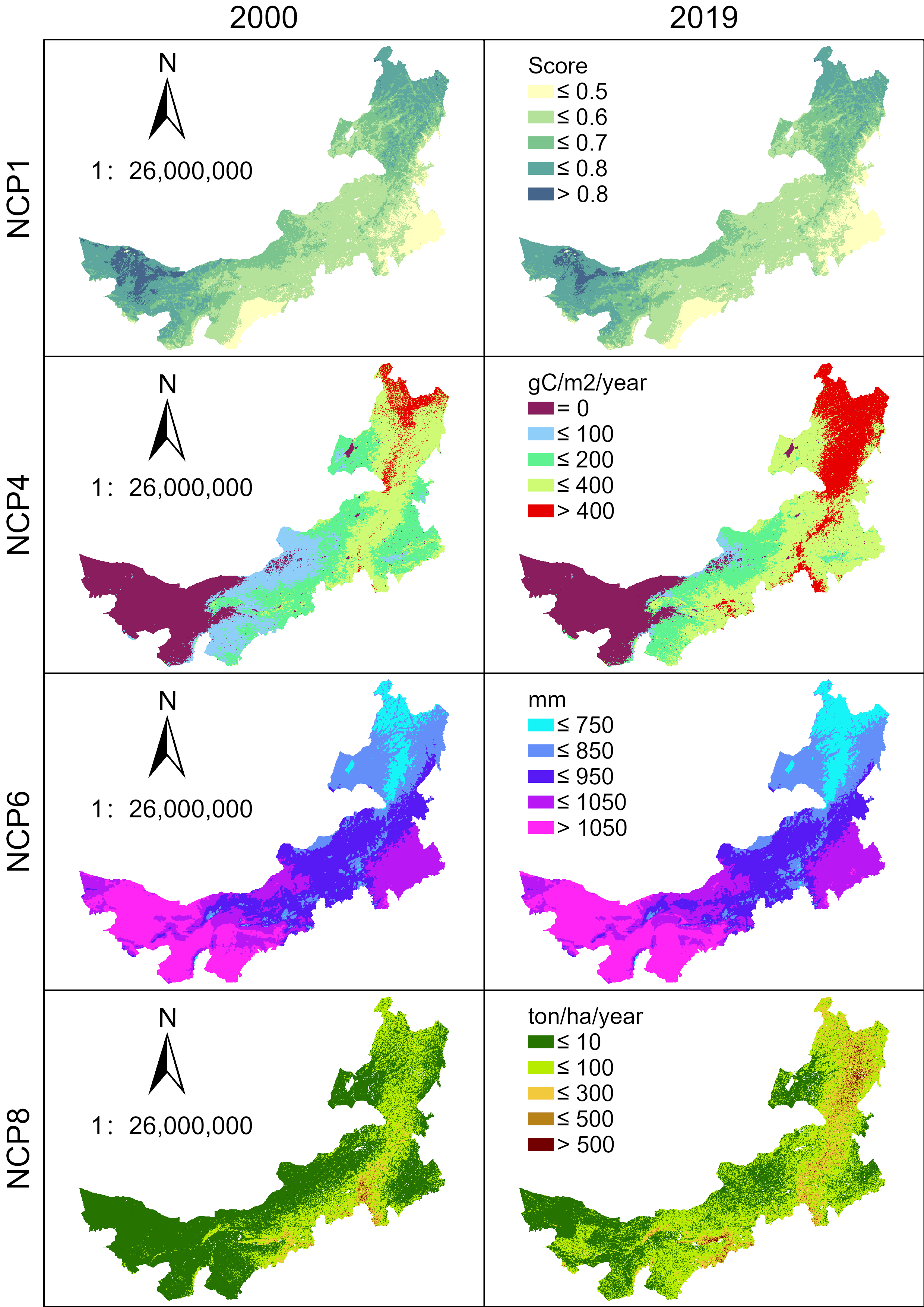


Figure S3. The spatial distribution of six drivers in Nei Mongol in 2000 and 2019.


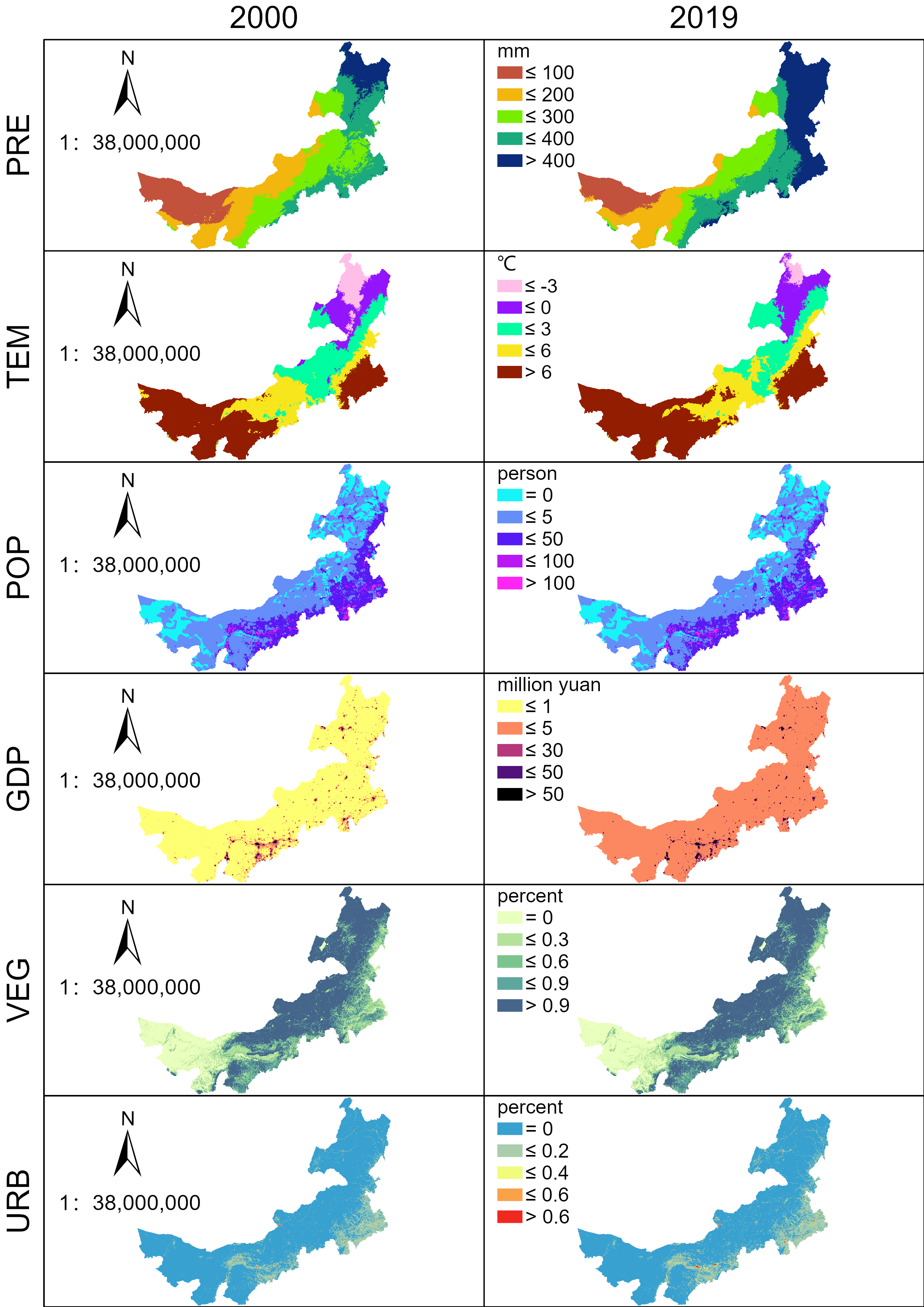


Table S1. The information on counties of Nei Mongol including name, area, abbreviation, Chinese name, and sample size.

| **City** | **Nr.** | **County** | **Area** | **Chinese name** | **Sample size** |
| --- | --- | --- | --- | --- | --- |
| Alashan  阿拉善盟 | 1 | Alashanyou | 72319.44 | 阿拉善右旗 | 993 |
|  | 2 | Alashanzuo | 80351.11 | 阿拉善左旗 | 990 |
|  | 3 | Ejina | 89889.78 | 额济纳旗 | 987 |
| Baotou  包头市 | 4 | Baiyunebokuang | 331.39 | 白云鄂博矿区 | 233 |
|  | 5 | Daerhanmaominganlianhe | 17518.63 | 达尔罕茂明安联合旗 | 992 |
|  | 6 | Donghe | 465.04 | 东河区 | 259 |
|  | 7 | Guyang | 4884.75 | 固阳县 | 996 |
|  | 8 | Jiuyuan | 819.53 | 九原区 | 452 |
|  | 9 | Kundulun | 288.57 | 昆都仑区 | 115 |
|  | 10 | Qingshan | 280.19 | 青山区 | 154 |
|  | 11 | Shiguai | 771.95 | 石拐区 | 540 |
|  | 12 | Tumoteyou | 2354.88 | 土默特右旗 | 975 |
| Bayannaoer  巴彦淖尔市 | 13 | Dengkou | 3687.07 | 磴口县 | 969 |
|  | 14 | Hangjinhou | 1749.32 | 杭锦后旗 | 965 |
|  | 15 | Linhe | 2362.40 | 临河区 | 946 |
|  | 16 | Wulatehou | 24655.18 | 乌拉特后旗 | 906 |
|  | 17 | Wulateqian | 7446.98 | 乌拉特前旗 | 927 |
|  | 18 | Wulatezhong | 22760.88 | 乌拉特中旗 | 981 |
|  | 19 | Wuyuan | 2506.15 | 五原县 | 967 |
| Chifeng  赤峰市 | 20 | Alukeerqin | 13394.51 | 阿鲁科尔沁旗 | 992 |
|  | 21 | Aohan | 8347.24 | 敖汉旗 | 982 |
|  | 22 | Balinyou | 10067.99 | 巴林右旗 | 981 |
|  | 23 | Balinzuo | 6536.44 | 巴林左旗 | 993 |
|  | 24 | Hongshan | 493.48 | 红山区 | 289 |
|  | 25 | Kalaqin | 3065.83 | 喀喇沁旗 | 990 |
|  | 26 | Keshiketeng | 19021.67 | 克什克腾旗 | 971 |
|  | 27 | Linxi | 3805.14 | 林西县 | 991 |
|  | 28 | Ningcheng | 4352.77 | 宁城县 | 989 |
|  | 29 | Songshan | 5675.06 | 松山区 | 977 |
|  | 30 | Wengniute | 12029.64 | 翁牛特旗 | 979 |
|  | 31 | Yuanbaoshan | 951.86 | 元宝山区 | 606 |
| Eerduosi  鄂尔多斯市 | 32 | Dalate | 8240.08 | 达拉特旗 | 988 |
|  | 33 | Dongsheng | 2165.44 | 东胜区 | 960 |
|  | 34 | Etuoke | 20389.42 | 鄂托克旗 | 994 |
|  | 35 | Etuokeqian | 12228.69 | 鄂托克前旗 | 994 |
|  | 36 | Hangjin | 18836.17 | 杭锦旗 | 993 |
|  | 37 | Kangbashi | 358.44 | 康巴什区 | 239 |
|  | 38 | Wushen | 11675.92 | 乌审旗 | 983 |
|  | 39 | Yijinhuoluo | 5461.60 | 伊金霍洛旗 | 981 |
|  | 40 | Zhungeer | 7552.30 | 准格尔旗 | 990 |
| Huhehaote  呼和浩特市 | 41 | Helingeer | 3443.51 | 和林格尔县 | 987 |
|  | 42 | Huimin | 194.71 | 回民区 | 107 |
|  | 43 | Qingshuihe | 2812.80 | 清水河县 | 988 |
|  | 44 | Saihan | 1002.35 | 赛罕区 | 597 |
|  | 45 | Tumotezuo | 2764.41 | 土默特左旗 | 942 |
|  | 46 | Tuoketuo | 1406.16 | 托克托县 | 950 |
|  | 47 | Wuchuan | 4677.65 | 武川县 | 997 |
|  | 48 | Xincheng | 659.86 | 新城区 | 405 |
|  | 49 | Yuquan | 205.20 | 玉泉区 | 104 |
| Hulunbeier  呼伦贝尔市 | 50 | Arong | 11283.95 | 阿荣旗 | 966 |
|  | 51 | Chenbaerhu | 17603.90 | 陈巴尔虎旗 | 948 |
|  | 52 | Eerguna | 29249.25 | 额尔古纳市 | 972 |
|  | 53 | Elunchun | 55770.81 | 鄂伦春自治旗 | 989 |
|  | 54 | Ewenkezu | 18830.26 | 鄂温克族自治旗 | 972 |
|  | 55 | Genhe | 20271.83 | 根河市 | 992 |
|  | 56 | Hailaer | 1326.80 | 海拉尔区 | 855 |
|  | 57 | Manzhouli | 481.31 | 满洲里市 | 172 |
|  | 58 | Molidawadawoerzu | 10586.28 | 莫力达瓦达斡尔族自治旗 | 960 |
|  | 59 | Xinbaerhuyou | 24973.29 | 新巴尔虎右旗 | 857 |
|  | 60 | Xinbaerhuzuo | 20290.45 | 新巴尔虎左旗 | 917 |
|  | 61 | Yakeshi | 28188.93 | 牙克石市 | 997 |
|  | 62 | Zhalainuoer | 268.14 | 扎赉诺尔区 | 140 |
|  | 63 | Zhalantun | 17048.68 | 扎兰屯市 | 994 |
| Tongliao  通辽市 | 64 | Huolinguole | 589.54 | 霍林郭勒市 | 386 |
|  | 65 | Kailu | 4427.80 | 开鲁县 | 966 |
|  | 66 | Keerqin | 3574.93 | 科尔沁区 | 952 |
|  | 67 | Keerqinzuoyihou | 11760.03 | 科尔沁左翼后旗 | 984 |
|  | 68 | Keerqinzuoyizhong | 9769.58 | 科尔沁左翼中旗 | 981 |
|  | 69 | Kulun | 4789.76 | 库伦旗 | 990 |
|  | 70 | Naiman | 8255.59 | 奈曼旗 | 978 |
|  | 71 | Zhalute | 16713.37 | 扎鲁特旗 | 988 |
| Wuhai  乌海市 | 72 | Haibowan | 484.05 | 海勃湾区 | 293 |
|  | 73 | Hainan | 967.65 | 海南区 | 643 |
|  | 74 | Wuda | 207.61 | 乌达区 | 99 |
| Wulanchabu  乌兰察布市 | 75 | Chaharrightfrontbanner | 2735.46 | 察哈尔右翼前旗 | 930 |
|  | 76 | Chaharrightmiddlebanner | 4186.99 | 察哈尔右翼中旗 | 997 |
|  | 77 | Chaharrightrearbanner | 3784.93 | 察哈尔右翼后旗 | 976 |
|  | 78 | Fengzhen | 2720.83 | 丰镇市 | 983 |
|  | 79 | Huade | 2536.80 | 化德县 | 982 |
|  | 80 | Jining | 114.21 | 集宁区 | 52 |
|  | 81 | Liangcheng | 3450.20 | 凉城县 | 945 |
|  | 82 | Shangdu | 4283.90 | 商都县 | 971 |
|  | 83 | Siziwang | 24016.92 | 四子王旗 | 994 |
|  | 84 | Xinghe | 3513.98 | 兴和县 | 980 |
|  | 85 | Zhuozi | 3095.73 | 卓资县 | 999 |
| Xilinguole  锡林郭勒盟 | 86 | Abaga | 27539.96 | 阿巴嘎旗 | 986 |
|  | 87 | Dongwuzhumuqin | 45820.21 | 东乌珠穆沁旗 | 936 |
|  | 88 | Duolun | 3881.45 | 多伦县 | 989 |
|  | 89 | Erlianhaote | 4023.28 | 二连浩特市 | 934 |
|  | 90 | Suniteyou | 22455.94 | 苏尼特右旗 | 994 |
|  | 91 | Sunitezuo | 34263.82 | 苏尼特左旗 | 963 |
|  | 92 | Taipusi | 3434.34 | 太仆寺旗 | 964 |
|  | 93 | Xianghuang | 5140.46 | 镶黄旗 | 997 |
|  | 94 | Xilinhaote | 14830.49 | 锡林浩特市 | 983 |
|  | 95 | Xiwuzhumuqin | 22606.86 | 西乌珠穆沁旗 | 993 |
|  | 96 | Zhenglan | 10238.85 | 正蓝旗 | 985 |
|  | 97 | Zhengxiangbai | 6264.56 | 正镶白旗 | 985 |
| Xing'an  兴安盟 | 98 | Aershan | 7501.95 | 阿尔山市 | 951 |
|  | 99 | Keerqinyouyiqian | 17017.34 | 科尔沁右翼前旗 | 991 |
|  | 100 | Keerqinyouyizhong | 12981.20 | 科尔沁右翼中旗 | 982 |
|  | 101 | Tuquan | 4876.53 | 突泉县 | 976 |
|  | 102 | Wulanhaote | 2336.44 | 乌兰浩特市 | 951 |
|  | 103 | Zhalaite | 11324.66 | 扎赉特旗 | 975 |

Table S2. The information on absolute value of impact of six drivers on four potential NCP in each county in Nei Mongol (values marked by yellow were used as examples in the section 3.3).

| County | PRE->HAB | TEM->HAB | POP->HAB | GDP->HAB | VEG->HAB | URB->HAB | PRE->NPP | TEM->NPP | POP->NPP | GDP->NPP | VEG->NPP | URB->NPP | PRE->PET | TEM->PET | POP->PET | GDP->PET | VEG->PET | URB->PET | PRE->SCC | TEM->SCC | POP->SCC | GDP->SCC | VEG->SCC | URB->SCC |
| --- | --- | --- | --- | --- | --- | --- | --- | --- | --- | --- | --- | --- | --- | --- | --- | --- | --- | --- | --- | --- | --- | --- | --- | --- |
| Abaga | -0.25 | 0.03 | -0.01 | -0.01 | 0.04 | -0.02 | -0.01 | -0.23 | 0.05 | 0.00 | 0.04 | -0.07 | 0.23 | 0.45 | 0.03 | 0.01 | -0.07 | 0.04 | 0.10 | 0.45 | 0.02 | 0.01 | 0.03 | -0.02 |
| Aershan | 0.16 | 0.03 | -0.03 | 0.01 | 0.13 | 0.13 | 0.46 | 0.01 | -0.02 | 0.00 | 0.01 | -0.05 | 0.76 | 0.14 | -0.01 | -0.02 | 0.01 | 0.06 | 0.26 | -0.05 | -0.02 | 0.02 | -0.02 | -0.06 |
| Alashanyou | 0.02 | 0.05 | 0.02 | 0.01 | 0.00 | -0.05 | 0.70 | 0.03 | -0.03 | -0.04 | 0.09 | 0.00 | -0.68 | -0.16 | -0.03 | -0.02 | 0.08 | -0.14 | 0.02 | 0.01 | -0.04 | 0.05 | 0.01 | -0.46 |
| Alashanzuo | 0.27 | 0.31 | 0.06 | 0.12 | 0.13 | -0.04 | 0.24 | 0.20 | -0.06 | 0.14 | 0.01 | 0.02 | -0.12 | 0.53 | 0.04 | -0.01 | -0.09 | 0.05 | 0.15 | 0.01 | 0.10 | 0.11 | 0.09 | -0.09 |
| Alukeerqin | -0.59 | -0.09 | -0.05 | 0.00 | 0.12 | -0.05 | 0.11 | 0.00 | -0.06 | -0.10 | -0.16 | -0.01 | 0.57 | 0.17 | 0.00 | -0.02 | 0.04 | 0.02 | -0.25 | 0.05 | -0.02 | 0.01 | -0.03 | -0.10 |
| Aohan | 0.18 | -0.20 | -0.07 | -0.01 | -0.14 | -0.01 | 0.03 | -0.18 | 0.00 | -0.08 | -0.01 | -0.23 | 0.87 | 0.24 | 0.03 | -0.06 | -0.02 | 0.05 | -0.02 | -0.11 | -0.05 | 0.01 | 0.00 | -0.06 |
| Arong | 0.67 | 0.00 | -0.05 | 0.03 | 0.08 | 0.12 | -0.55 | -0.04 | 0.03 | 0.02 | -0.16 | -0.09 | -0.39 | 0.11 | 0.03 | -0.01 | 0.01 | 0.00 | -0.47 | -0.06 | 0.01 | -0.02 | -0.14 | -0.08 |
| Baiyunebokuang | -0.20 | -0.12 | 0.03 | 0.47 | -0.13 | -0.17 | 0.09 | 0.01 | -0.01 | -0.21 | 0.52 | -0.27 | 0.44 | 0.22 | 0.07 | -0.01 | 0.05 | 0.15 | -0.08 | -0.09 | 0.08 | 0.20 | 0.00 | 0.01 |
| Balinyou | 0.07 | 0.01 | 0.06 | -0.10 | 0.14 | -0.13 | 0.24 | -0.06 | 0.03 | 0.04 | -0.06 | 0.10 | 0.42 | 0.17 | 0.03 | 0.02 | 0.04 | -0.06 | 0.46 | 0.06 | 0.00 | 0.02 | 0.02 | 0.04 |
| Balinzuo | 0.03 | -0.05 | -0.11 | -0.23 | -0.15 | -0.25 | 0.19 | 0.04 | -0.02 | -0.08 | 0.11 | -0.05 | -0.38 | -0.10 | 0.05 | 0.04 | 0.18 | 0.10 | 0.38 | 0.21 | -0.06 | -0.02 | -0.23 | -0.11 |
| Chaharrightfrontbanner | 0.10 | -0.03 | -0.05 | -0.17 | 0.24 | -0.14 | 0.04 | 0.05 | -0.05 | -0.24 | 0.05 | -0.28 | -0.37 | -0.28 | -0.05 | 0.07 | 0.04 | 0.03 | -0.23 | -0.10 | -0.01 | -0.06 | 0.13 | -0.07 |
| Chaharrightmiddlebanner | 0.64 | 0.06 | 0.03 | 0.01 | 0.10 | 0.03 | 0.41 | 0.12 | 0.04 | 0.05 | -0.12 | -0.11 | 0.63 | 0.25 | -0.04 | -0.02 | -0.06 | 0.04 | 0.44 | 0.17 | 0.01 | 0.00 | -0.16 | -0.01 |
| Chaharrightrearbanner | 0.10 | -0.33 | -0.01 | -0.03 | -0.26 | 0.00 | 0.02 | -0.28 | -0.07 | -0.08 | -0.29 | -0.03 | 0.33 | 0.04 | 0.00 | -0.08 | -0.30 | 0.00 | 0.35 | 0.05 | -0.02 | -0.05 | 0.13 | 0.03 |
| Chenbaerhu | -0.58 | 0.05 | -0.05 | 0.04 | -0.04 | 0.03 | 0.20 | -0.05 | 0.05 | -0.03 | 0.00 | 0.01 | 0.36 | -0.27 | 0.01 | 0.04 | -0.05 | 0.02 | 0.51 | -0.05 | 0.01 | -0.04 | 0.02 | -0.03 |
| Daerhanmaominganlianhe | 0.90 | -0.05 | -0.01 | -0.01 | 0.04 | 0.03 | 0.49 | 0.38 | -0.03 | 0.00 | -0.11 | -0.05 | 0.85 | 0.19 | -0.01 | 0.01 | 0.02 | 0.01 | 0.04 | -0.24 | 0.14 | 0.26 | -0.03 | -0.01 |
| Dalate | 0.68 | -0.25 | 0.03 | -0.02 | 0.08 | -0.26 | 0.18 | 0.08 | -0.04 | 0.10 | -0.35 | -0.10 | -0.21 | 0.14 | -0.01 | -0.03 | -0.05 | 0.14 | 0.30 | -0.18 | 0.00 | -0.03 | 0.10 | -0.10 |
| Dengkou | -0.03 | -0.04 | 0.01 | -0.01 | -0.09 | -0.09 | 0.35 | 0.22 | -0.01 | 0.03 | -0.33 | 0.11 | 0.36 | 0.05 | 0.00 | 0.02 | 0.14 | 0.23 | -0.19 | -0.03 | 0.00 | 0.01 | -0.13 | -0.07 |
| Donghe | 0.25 | 0.24 | -0.06 | 0.09 | 0.07 | -0.46 | 0.19 | 0.08 | 0.06 | -0.28 | 0.03 | -0.28 | -0.26 | -0.02 | 0.10 | 0.15 | 0.03 | 0.36 | 0.25 | 0.32 | -0.04 | 0.03 | 0.01 | -0.29 |
| Dongsheng | 0.78 | 0.11 | 0.01 | -0.06 | -0.24 | -0.07 | 0.27 | -0.05 | -0.09 | 0.05 | 0.04 | -0.13 | 0.32 | 0.24 | 0.04 | -0.42 | -0.26 | 0.08 | 0.21 | 0.09 | 0.03 | -0.10 | -0.26 | -0.10 |
| Dongwuzhumuqin | 0.12 | 0.07 | 0.02 | 0.03 | -0.14 | -0.09 | 0.40 | 0.16 | -0.02 | 0.03 | -0.01 | 0.01 | -0.64 | -0.24 | 0.02 | -0.01 | 0.02 | 0.06 | 0.26 | 0.12 | -0.02 | -0.04 | -0.01 | -0.02 |
| Duolun | -0.13 | -0.19 | -0.05 | -0.18 | -0.15 | -0.09 | -0.07 | -0.12 | -0.05 | -0.01 | 0.13 | -0.22 | 0.02 | -0.43 | 0.06 | -0.01 | 0.25 | 0.04 | 0.12 | 0.10 | -0.01 | 0.04 | 0.08 | 0.04 |
| Eerguna | -0.46 | -0.07 | 0.07 | 0.13 | -0.02 | 0.13 | -0.18 | -0.01 | -0.01 | -0.08 | 0.05 | -0.07 | 0.25 | 0.01 | -0.05 | -0.08 | 0.02 | -0.09 | 0.25 | 0.12 | -0.16 | -0.10 | -0.01 | -0.11 |
| Ejina | 0.47 | -0.44 | -0.13 | 0.07 | 0.04 | -0.06 | -0.02 | -0.05 | -0.05 | -0.09 | -0.30 | 0.12 | -0.35 | 0.04 | -0.04 | -0.04 | -0.06 | 0.05 | 0.21 | -0.17 | 0.01 | -0.02 | 0.00 | 0.00 |
| Elunchun | 0.60 | 0.10 | 0.04 | 0.00 | 0.01 | 0.12 | 0.28 | 0.15 | -0.03 | 0.03 | 0.08 | -0.14 | 0.43 | 0.43 | -0.01 | -0.03 | 0.07 | 0.00 | 0.18 | 0.22 | -0.04 | 0.03 | 0.03 | -0.08 |
| Erlianhaote | 0.76 | 0.58 | 0.02 | 0.07 | 0.05 | 0.07 | -0.10 | -0.07 | 0.01 | 0.14 | 0.15 | -0.01 | -0.40 | -0.55 | -0.03 | -0.09 | -0.01 | -0.01 | 0.08 | 0.04 | 0.05 | 0.02 | 0.07 | -0.06 |
| Etuoke | 0.76 | 0.11 | 0.02 | 0.06 | 0.10 | -0.06 | 0.67 | -0.19 | -0.01 | -0.04 | -0.07 | 0.01 | 0.50 | 0.46 | 0.03 | -0.02 | -0.05 | -0.01 | -0.12 | -0.03 | 0.01 | 0.07 | 0.00 | -0.06 |
| Etuokeqian | 0.12 | -0.31 | 0.26 | -0.03 | 0.02 | -0.26 | -0.01 | -0.20 | -0.11 | 0.06 | -0.35 | 0.06 | 0.00 | -0.11 | 0.28 | -0.02 | -0.11 | -0.25 | 0.05 | -0.15 | 0.13 | -0.03 | 0.19 | -0.11 |
| Ewenkezu | -0.83 | -0.05 | -0.02 | 0.02 | 0.00 | 0.00 | 0.18 | 0.06 | -0.09 | -0.09 | 0.10 | -0.03 | -0.07 | -0.05 | 0.01 | 0.06 | -0.01 | 0.03 | 0.57 | -0.05 | -0.06 | -0.06 | 0.01 | -0.01 |
| Fengzhen | 0.21 | 0.10 | 0.00 | -0.22 | -0.09 | -0.12 | -0.06 | 0.01 | -0.06 | -0.37 | 0.02 | -0.24 | -0.78 | -0.13 | -0.06 | 0.04 | -0.11 | 0.12 | -0.14 | 0.01 | 0.01 | -0.08 | -0.22 | -0.08 |
| Genhe | -0.35 | 0.05 | 0.01 | 0.00 | -0.02 | 0.09 | -0.48 | -0.05 | 0.06 | 0.02 | 0.02 | -0.03 | -0.35 | -0.19 | -0.06 | -0.12 | -0.06 | -0.05 | 0.20 | 0.03 | 0.03 | 0.00 | 0.00 | -0.10 |
| Guyang | 0.41 | 0.14 | -0.04 | -0.05 | -0.24 | -0.09 | 0.22 | -0.01 | -0.03 | -0.12 | -0.24 | -0.10 | 0.21 | 0.19 | 0.01 | 0.07 | -0.13 | 0.07 | 0.20 | 0.08 | -0.04 | -0.03 | -0.27 | -0.05 |
| Haibowan | 0.57 | 0.49 | -0.04 | -0.30 | -0.20 | -0.19 | 0.20 | 0.12 | -0.06 | -0.19 | 0.04 | 0.34 | 0.15 | 0.16 | 0.00 | 0.04 | -0.01 | 0.08 | 0.21 | 0.20 | 0.07 | -0.12 | -0.10 | -0.21 |
| Hailaer | -0.45 | -0.12 | 0.02 | 0.01 | 0.17 | -0.08 | 0.00 | 0.00 | -0.02 | -0.35 | -0.10 | -0.36 | -0.04 | -0.19 | -0.03 | 0.15 | -0.09 | 0.06 | 0.40 | 0.01 | -0.02 | 0.01 | 0.05 | 0.00 |
| Hainan | -0.04 | -0.27 | -0.01 | -0.05 | -0.13 | -0.25 | 0.50 | 0.03 | -0.08 | -0.20 | 0.00 | 0.09 | 0.13 | -0.07 | 0.05 | 0.23 | -0.02 | -0.18 | -0.30 | -0.09 | -0.02 | 0.06 | -0.01 | -0.18 |
| Hangjin | 0.85 | 0.14 | 0.02 | 0.00 | 0.00 | -0.02 | 0.71 | -0.03 | 0.06 | 0.02 | -0.02 | 0.11 | 0.19 | 0.12 | -0.03 | -0.04 | 0.26 | 0.05 | 0.19 | 0.00 | -0.07 | -0.03 | 0.03 | -0.05 |
| Hangjinhou | 0.18 | -0.08 | 0.03 | 0.13 | -0.19 | 0.02 | 0.00 | 0.05 | -0.06 | -0.03 | -0.43 | -0.05 | 0.42 | -0.18 | 0.03 | 0.03 | -0.13 | 0.01 | -0.09 | -0.03 | -0.02 | -0.04 | 0.20 | -0.01 |
| Helingeer | 0.18 | 0.09 | -0.03 | -0.08 | 0.34 | -0.09 | 0.27 | 0.04 | 0.00 | -0.02 | -0.18 | -0.08 | -0.21 | -0.02 | 0.01 | 0.09 | -0.26 | 0.05 | 0.14 | 0.12 | -0.03 | 0.02 | 0.26 | -0.08 |
| Hongshan | 0.00 | 0.03 | -0.04 | -0.69 | -0.13 | -0.28 | -0.09 | -0.15 | 0.04 | -0.23 | 0.22 | -0.44 | 0.74 | 0.40 | -0.06 | -0.29 | -0.07 | 0.01 | 0.21 | 0.17 | 0.02 | -0.02 | -0.05 | 0.03 |
| Huade | 0.34 | -0.07 | -0.12 | -0.07 | -0.16 | -0.04 | -0.04 | 0.05 | -0.03 | 0.17 | -0.12 | -0.23 | 0.56 | -0.03 | -0.02 | 0.00 | -0.03 | 0.03 | -0.06 | 0.05 | 0.06 | 0.01 | 0.16 | 0.02 |
| Huimin | -0.42 | -0.18 | -0.01 | -0.38 | 0.10 | -0.68 | -0.01 | -0.03 | 0.17 | -0.38 | -0.01 | -0.76 | -0.72 | -0.25 | -0.02 | 0.35 | 0.27 | -0.02 | -0.40 | -0.17 | 0.06 | -0.57 | 0.04 | -0.11 |
| Huolinguole | 0.05 | 0.06 | 0.02 | 0.22 | -0.35 | -0.28 | 0.17 | 0.03 | -0.10 | -0.11 | -0.06 | -0.54 | 0.67 | 0.15 | 0.05 | 0.42 | 0.02 | 0.23 | 0.14 | 0.08 | -0.07 | -0.16 | -0.19 | -0.22 |
| Jining | -0.06 | -0.28 | 0.27 | -0.18 | 0.00 | -0.36 | 0.06 | -0.08 | 0.52 | 0.08 | -0.48 | -0.30 | -0.34 | -0.20 | 0.17 | 0.24 | 0.16 | -0.17 | -0.17 | -0.05 | -0.02 | -0.05 | 0.08 | 0.18 |
| Jiuyuan | 0.56 | 0.04 | -0.02 | -0.28 | 0.07 | -0.24 | 0.22 | -0.05 | -0.03 | -0.13 | -0.07 | -0.31 | -0.58 | 0.01 | 0.07 | 0.20 | -0.22 | -0.11 | 0.50 | 0.02 | -0.02 | -0.27 | 0.12 | -0.18 |
| Kailu | 0.55 | 0.23 | -0.02 | -0.04 | -0.04 | -0.01 | -0.06 | -0.04 | -0.06 | -0.03 | 0.07 | -0.09 | -0.20 | 0.65 | 0.05 | 0.03 | 0.06 | -0.02 | -0.23 | -0.01 | -0.02 | 0.08 | -0.02 | -0.02 |
| Kalaqin | -0.18 | -0.07 | -0.01 | -0.02 | -0.04 | -0.17 | -0.15 | -0.07 | -0.01 | 0.04 | -0.03 | -0.46 | 0.88 | 0.34 | -0.02 | -0.05 | -0.09 | -0.01 | 0.59 | 0.30 | 0.01 | -0.01 | 0.08 | 0.09 |
| Kangbashi | 0.17 | 0.13 | 0.12 | -0.43 | 0.04 | -0.14 | -0.26 | -0.22 | 0.06 | 0.36 | -0.22 | -0.30 | 0.34 | 0.26 | 0.02 | -0.02 | 0.09 | 0.08 | -0.15 | 0.10 | -0.02 | 0.03 | 0.07 | -0.06 |
| Keerqin | 0.25 | -0.13 | -0.01 | -0.07 | -0.03 | 0.08 | 0.25 | 0.13 | -0.01 | -0.11 | 0.10 | -0.16 | 0.52 | 0.46 | -0.01 | -0.01 | 0.00 | -0.02 | -0.29 | -0.44 | -0.01 | -0.07 | 0.03 | 0.01 |
| Keerqinyouyiqian | 0.51 | 0.05 | -0.02 | -0.13 | -0.11 | 0.07 | 0.67 | 0.08 | 0.06 | -0.02 | 0.05 | -0.14 | 0.67 | 0.09 | 0.03 | -0.01 | -0.09 | 0.06 | -0.32 | -0.02 | -0.03 | 0.03 | 0.15 | -0.11 |
| Keerqinyouyizhong | -0.45 | -0.10 | 0.04 | -0.03 | 0.08 | -0.10 | -0.06 | -0.07 | -0.03 | -0.05 | 0.00 | -0.10 | 0.83 | 0.09 | -0.03 | -0.01 | 0.00 | 0.06 | -0.45 | -0.07 | 0.00 | 0.01 | 0.07 | -0.09 |
| Keerqinzuoyihou | -0.04 | -0.05 | -0.07 | 0.01 | -0.02 | -0.13 | -0.07 | 0.25 | -0.06 | -0.05 | 0.09 | -0.01 | 0.68 | 0.22 | 0.00 | -0.03 | -0.03 | 0.06 | -0.16 | -0.30 | 0.00 | 0.08 | -0.01 | -0.03 |
| Keerqinzuoyizhong | 0.35 | -0.46 | -0.04 | -0.02 | 0.06 | -0.01 | 0.25 | -0.06 | 0.00 | 0.09 | 0.08 | -0.12 | 0.72 | 0.17 | 0.01 | 0.01 | -0.07 | 0.01 | -0.63 | -0.14 | -0.01 | 0.01 | 0.12 | -0.03 |
| Keshiketeng | 0.24 | 0.04 | 0.00 | -0.04 | 0.00 | -0.02 | 0.11 | 0.04 | 0.00 | -0.01 | 0.08 | -0.13 | 0.35 | 0.23 | -0.01 | -0.03 | 0.02 | 0.06 | -0.15 | 0.13 | 0.01 | -0.04 | -0.02 | 0.06 |
| Kulun | -0.22 | -0.26 | -0.02 | -0.01 | -0.01 | -0.02 | -0.05 | -0.11 | 0.00 | 0.01 | 0.37 | 0.01 | 0.32 | 0.11 | 0.00 | 0.03 | -0.04 | 0.19 | 0.03 | -0.14 | 0.00 | 0.00 | 0.00 | 0.01 |
| Kundulun | 0.25 | 0.17 | -0.02 | -0.45 | 0.04 | -0.30 | 0.17 | -0.14 | 0.04 | -0.21 | -0.04 | -0.26 | -0.36 | 0.00 | 0.00 | -0.09 | 0.18 | -0.02 | 0.24 | -0.03 | -0.01 | -0.34 | 0.02 | -0.19 |
| Liangcheng | 0.22 | 0.06 | -0.02 | -0.05 | 0.25 | -0.14 | 0.52 | 0.18 | 0.01 | -0.06 | -0.02 | -0.17 | -0.65 | -0.17 | -0.01 | 0.00 | -0.05 | 0.03 | 0.49 | 0.22 | 0.01 | -0.03 | -0.15 | -0.09 |
| Linhe | -0.12 | 0.04 | 0.00 | 0.06 | 0.04 | 0.14 | -0.13 | -0.10 | 0.03 | 0.13 | -0.30 | -0.18 | 0.03 | 0.05 | 0.00 | 0.05 | 0.15 | 0.15 | 0.11 | -0.04 | -0.01 | -0.03 | 0.03 | 0.01 |
| Linxi | 0.05 | -0.21 | -0.06 | -0.13 | -0.12 | -0.11 | 0.11 | -0.04 | -0.03 | 0.00 | -0.05 | -0.22 | -0.09 | 0.06 | 0.05 | 0.04 | -0.27 | -0.18 | 0.40 | 0.22 | 0.05 | 0.04 | 0.04 | 0.02 |
| Manzhouli | -0.10 | -0.11 | -0.01 | 0.24 | 0.02 | 0.06 | 0.04 | -0.18 | -0.03 | -0.13 | 0.11 | -0.19 | 0.05 | 0.59 | 0.00 | -0.02 | 0.06 | 0.05 | 0.06 | -0.13 | -0.01 | -0.05 | 0.01 | -0.10 |
| Molidawadawoerzu | 0.33 | -0.16 | -0.03 | -0.02 | 0.18 | 0.20 | 0.13 | -0.06 | 0.07 | 0.02 | 0.10 | -0.15 | 0.32 | -0.19 | -0.05 | 0.03 | -0.04 | -0.03 | -0.08 | 0.28 | -0.04 | 0.01 | 0.02 | -0.11 |
| Naiman | 0.25 | -0.49 | -0.06 | 0.01 | 0.15 | -0.05 | 0.26 | -0.03 | 0.03 | 0.00 | -0.06 | -0.06 | 0.19 | 0.06 | 0.00 | 0.01 | 0.10 | 0.03 | 0.05 | -0.20 | -0.07 | 0.00 | 0.06 | -0.05 |
| Ningcheng | -0.01 | 0.00 | -0.02 | -0.03 | -0.07 | -0.03 | -0.51 | -0.31 | -0.04 | -0.09 | -0.02 | -0.31 | 0.85 | 0.53 | -0.01 | 0.03 | 0.00 | 0.09 | 0.18 | 0.12 | 0.02 | 0.03 | 0.04 | 0.20 |
| Qingshan | 0.07 | -0.05 | -0.10 | -0.71 | -0.37 | -0.21 | 0.01 | -0.01 | 0.05 | -0.46 | -0.24 | -0.15 | -0.50 | -0.05 | 0.14 | 0.09 | 0.08 | -0.22 | 0.04 | -0.04 | -0.08 | -0.37 | -0.23 | -0.13 |
| Qingshuihe | 0.37 | 0.20 | 0.01 | 0.09 | -0.05 | -0.05 | -0.02 | -0.13 | 0.05 | -0.06 | 0.06 | -0.26 | 0.20 | 0.19 | 0.04 | 0.11 | -0.08 | 0.06 | 0.12 | 0.07 | 0.05 | 0.04 | 0.08 | -0.09 |
| Saihan | 0.42 | 0.35 | -0.06 | -0.13 | 0.17 | -0.10 | 0.25 | 0.10 | 0.07 | -0.32 | -0.03 | -0.37 | -0.42 | -0.15 | -0.07 | 0.30 | -0.15 | 0.21 | 0.27 | 0.32 | -0.05 | -0.06 | 0.05 | -0.08 |
| Shangdu | 0.10 | 0.00 | 0.00 | -0.10 | 0.14 | 0.00 | -0.24 | -0.13 | -0.01 | -0.08 | -0.21 | -0.10 | 0.45 | 0.05 | -0.05 | 0.01 | -0.18 | 0.10 | -0.06 | 0.03 | -0.01 | 0.03 | -0.06 | 0.00 |
| Shiguai | 0.01 | -0.10 | -0.13 | -0.35 | -0.14 | -0.19 | 0.31 | 0.17 | -0.05 | -0.18 | -0.07 | -0.16 | -0.40 | 0.07 | 0.00 | 0.23 | -0.08 | 0.11 | 0.24 | 0.10 | -0.01 | -0.09 | -0.15 | -0.07 |
| Siziwang | 0.77 | 0.03 | -0.05 | -0.03 | 0.08 | 0.06 | 0.64 | 0.11 | -0.01 | 0.08 | -0.05 | 0.01 | 0.75 | 0.05 | -0.07 | 0.03 | 0.11 | 0.04 | 0.08 | 0.01 | -0.05 | -0.05 | -0.06 | 0.16 |
| Songshan | -0.56 | -0.24 | 0.02 | -0.09 | 0.07 | -0.24 | -0.23 | -0.09 | 0.03 | -0.06 | 0.07 | -0.15 | 0.93 | 0.28 | -0.01 | -0.01 | -0.01 | 0.04 | 0.40 | 0.17 | -0.02 | 0.01 | 0.06 | -0.02 |
| Suniteyou | -0.10 | -0.15 | 0.02 | -0.06 | 0.09 | -0.02 | 0.09 | -0.22 | -0.06 | 0.02 | -0.02 | -0.14 | 0.19 | -0.28 | 0.01 | 0.03 | 0.14 | -0.01 | -0.05 | -0.05 | -0.18 | -0.09 | -0.03 | -0.40 |
| Sunitezuo | 0.77 | 0.26 | 0.03 | 0.02 | -0.06 | -0.09 | 0.30 | -0.27 | 0.00 | -0.02 | -0.06 | -0.07 | 0.01 | 0.32 | 0.03 | -0.01 | -0.01 | -0.03 | -0.25 | 0.26 | 0.03 | 0.01 | 0.02 | -0.01 |
| Taipusi | 0.34 | 0.03 | -0.02 | 0.08 | 0.00 | -0.09 | 0.15 | -0.02 | 0.15 | 0.04 | -0.06 | -0.11 | -0.41 | -0.12 | -0.10 | -0.13 | -0.18 | 0.01 | -0.36 | 0.01 | 0.01 | -0.05 | 0.07 | 0.06 |
| Tumoteyou | 0.57 | 0.28 | -0.01 | -0.07 | -0.02 | -0.15 | 0.26 | 0.11 | -0.03 | 0.04 | -0.04 | -0.21 | -0.11 | 0.04 | 0.11 | 0.32 | 0.08 | -0.01 | 0.46 | 0.32 | 0.00 | 0.04 | -0.04 | -0.12 |
| Tumotezuo | 0.71 | 0.20 | -0.03 | 0.24 | 0.02 | -0.15 | 0.28 | 0.09 | 0.02 | 0.17 | -0.09 | -0.33 | -0.55 | -0.04 | -0.01 | 0.10 | -0.05 | 0.04 | 0.56 | 0.21 | -0.02 | 0.19 | 0.02 | -0.13 |
| Tuoketuo | 0.18 | 0.13 | -0.09 | 0.01 | 0.02 | 0.08 | 0.02 | 0.05 | 0.00 | -0.07 | -0.21 | -0.33 | -0.05 | 0.25 | -0.01 | -0.19 | 0.02 | 0.05 | 0.07 | 0.18 | 0.01 | -0.01 | 0.10 | -0.03 |
| Tuquan | -0.01 | -0.06 | -0.07 | -0.14 | 0.38 | -0.08 | 0.11 | -0.06 | 0.01 | -0.14 | 0.14 | -0.24 | 0.23 | 0.11 | 0.07 | 0.13 | -0.40 | 0.11 | -0.44 | -0.17 | -0.08 | -0.10 | 0.17 | -0.08 |
| Wengniute | -0.64 | -0.22 | -0.02 | -0.06 | 0.01 | -0.05 | -0.28 | 0.01 | 0.02 | -0.08 | 0.01 | -0.01 | 0.87 | 0.17 | -0.02 | 0.01 | 0.01 | -0.03 | -0.22 | 0.04 | -0.03 | -0.01 | 0.00 | -0.03 |
| Wuchuan | 0.20 | 0.06 | -0.01 | -0.06 | -0.21 | -0.02 | -0.09 | 0.05 | 0.05 | -0.08 | -0.45 | -0.03 | -0.19 | -0.02 | 0.03 | 0.06 | -0.07 | -0.05 | -0.05 | -0.04 | -0.03 | -0.03 | -0.23 | 0.04 |
| Wuda | -0.23 | -0.37 | -0.29 | 0.23 | -0.27 | -0.25 | -0.06 | 0.23 | 0.00 | -0.13 | -0.14 | 0.15 | 0.03 | 0.38 | -0.03 | -0.16 | 0.32 | 0.16 | -0.26 | 0.08 | -0.05 | 0.16 | -0.09 | -0.17 |
| Wulanhaote | 0.41 | 0.08 | 0.00 | 0.05 | -0.04 | -0.06 | 0.42 | -0.02 | 0.10 | 0.06 | 0.16 | -0.25 | -0.14 | -0.10 | -0.08 | -0.12 | 0.18 | -0.13 | -0.06 | -0.01 | 0.02 | 0.04 | -0.08 | -0.04 |
| Wulatehou | 0.52 | -0.08 | -0.08 | 0.00 | -0.02 | 0.01 | 0.32 | -0.09 | -0.42 | -0.01 | 0.13 | -0.03 | 0.19 | 0.40 | -0.02 | -0.04 | -0.11 | 0.04 | 0.37 | -0.20 | -0.16 | -0.01 | 0.03 | -0.04 |
| Wulateqian | 0.55 | 0.03 | -0.02 | 0.00 | 0.03 | 0.00 | 0.24 | 0.06 | -0.02 | 0.04 | -0.24 | -0.13 | -0.68 | -0.08 | 0.02 | 0.02 | -0.03 | -0.01 | 0.27 | -0.07 | 0.02 | -0.01 | 0.04 | 0.02 |
| Wulatezhong | 0.73 | 0.04 | -0.04 | -0.02 | -0.03 | 0.02 | 0.60 | 0.05 | -0.11 | 0.05 | -0.20 | 0.03 | 0.54 | 0.06 | -0.06 | 0.05 | -0.16 | 0.04 | 0.04 | 0.10 | 0.02 | -0.01 | -0.06 | 0.05 |
| Wushen | 0.71 | -0.28 | 0.02 | -0.01 | -0.06 | 0.01 | 0.19 | -0.03 | 0.11 | -0.08 | -0.33 | -0.12 | -0.14 | 0.98 | 0.00 | 0.01 | 0.06 | -0.01 | 0.10 | -0.09 | -0.02 | 0.04 | 0.19 | -0.04 |
| Wuyuan | -0.10 | -0.04 | 0.08 | -0.18 | -0.08 | 0.16 | 0.05 | -0.01 | -0.04 | 0.08 | -0.05 | -0.10 | 0.77 | 0.09 | 0.01 | 0.08 | 0.10 | 0.11 | -0.04 | 0.00 | 0.00 | 0.01 | 0.07 | 0.01 |
| Xianghuang | -0.36 | -0.18 | -0.13 | -0.10 | 0.16 | -0.01 | -0.47 | -0.15 | 0.01 | -0.01 | -0.04 | -0.05 | 0.40 | -0.07 | -0.03 | 0.02 | -0.11 | 0.02 | 0.28 | 0.14 | 0.08 | 0.07 | -0.10 | 0.01 |
| Xilinhaote | 0.12 | -0.21 | -0.07 | -0.20 | -0.03 | -0.06 | -0.12 | -0.33 | 0.06 | 0.01 | 0.02 | -0.23 | -0.41 | 0.38 | 0.04 | 0.05 | 0.10 | 0.09 | -0.29 | 0.13 | 0.01 | -0.03 | 0.08 | 0.05 |
| Xinbaerhuyou | 0.18 | 0.06 | -0.04 | -0.03 | 0.10 | -0.10 | 0.24 | -0.19 | -0.04 | 0.02 | -0.07 | -0.10 | -0.44 | 0.26 | -0.03 | -0.01 | -0.08 | 0.03 | 0.22 | 0.07 | -0.03 | -0.02 | -0.03 | -0.06 |
| Xinbaerhuzuo | -0.53 | 0.00 | 0.02 | -0.02 | 0.00 | -0.07 | -0.19 | 0.05 | -0.01 | 0.00 | 0.02 | -0.06 | -0.74 | -0.13 | -0.01 | 0.01 | 0.01 | 0.02 | 0.68 | 0.10 | -0.01 | 0.02 | 0.02 | 0.00 |
| Xincheng | 0.20 | 0.10 | -0.03 | -0.38 | -0.44 | -0.39 | 0.28 | 0.16 | 0.10 | -0.27 | -0.16 | -0.54 | -0.81 | -0.35 | -0.04 | 0.24 | -0.13 | 0.00 | -0.08 | 0.07 | -0.02 | -0.17 | -0.31 | -0.22 |
| Xinghe | -0.26 | -0.21 | -0.03 | -0.13 | 0.09 | -0.06 | -0.46 | -0.13 | 0.03 | -0.03 | -0.14 | -0.21 | -0.77 | -0.30 | -0.03 | -0.02 | 0.06 | 0.05 | -0.37 | -0.27 | 0.01 | -0.08 | -0.05 | -0.07 |
| Xiwuzhumuqin | 0.39 | -0.09 | -0.08 | -0.06 | 0.11 | 0.08 | 0.21 | -0.05 | -0.11 | -0.02 | 0.00 | -0.36 | -0.41 | -0.22 | 0.00 | 0.02 | -0.15 | -0.12 | 0.46 | -0.05 | 0.02 | -0.02 | 0.03 | -0.04 |
| Yakeshi | 0.23 | 0.21 | 0.00 | 0.14 | -0.12 | 0.02 | 0.34 | 0.00 | 0.04 | -0.03 | -0.01 | -0.08 | 0.71 | 0.14 | 0.01 | 0.08 | 0.03 | -0.03 | 0.45 | -0.08 | 0.03 | -0.13 | -0.01 | 0.11 |
| Yijinhuoluo | 0.63 | 0.17 | -0.01 | 0.07 | -0.08 | -0.04 | 0.31 | 0.05 | -0.07 | -0.11 | -0.35 | -0.28 | 0.50 | 0.13 | -0.02 | -0.03 | -0.06 | 0.06 | 0.25 | -0.01 | -0.08 | 0.03 | 0.00 | 0.01 |
| Yuanbaoshan | -0.20 | -0.37 | -0.05 | 0.04 | -0.04 | -0.08 | -0.08 | -0.20 | -0.02 | 0.01 | -0.06 | -0.41 | 0.22 | 0.32 | 0.00 | 0.10 | -0.27 | -0.07 | 0.19 | -0.05 | -0.03 | -0.09 | 0.09 | -0.05 |
| Yuquan | -0.07 | -0.02 | 0.23 | -0.24 | 0.06 | -0.11 | 0.14 | -0.19 | -0.02 | -0.39 | -0.08 | -0.38 | -0.37 | -0.10 | -0.17 | 0.02 | -0.05 | 0.00 | 0.12 | 0.11 | -0.08 | -0.09 | 0.12 | 0.34 |
| Zhalainuoer | 0.09 | 0.24 | -0.04 | 0.38 | 0.12 | 0.21 | -0.19 | -0.18 | 0.18 | -0.21 | -0.25 | -0.54 | 0.00 | 0.27 | -0.17 | -0.33 | 0.05 | 0.09 | -0.14 | -0.31 | 0.02 | -0.14 | -0.03 | -0.11 |
| Zhalaite | -0.12 | -0.10 | 0.00 | -0.08 | -0.10 | -0.10 | -0.03 | -0.14 | 0.00 | 0.03 | 0.08 | -0.10 | 0.64 | 0.23 | 0.02 | 0.02 | -0.03 | 0.02 | -0.47 | -0.10 | -0.01 | 0.00 | -0.01 | -0.09 |
| Zhalantun | 0.32 | 0.00 | -0.05 | -0.06 | 0.12 | 0.19 | 0.00 | -0.04 | -0.01 | -0.08 | -0.06 | -0.08 | -0.80 | -0.07 | 0.00 | -0.05 | -0.01 | -0.08 | -0.01 | 0.01 | 0.04 | 0.01 | -0.10 | -0.14 |
| Zhalute | -0.28 | 0.09 | -0.10 | -0.04 | 0.01 | -0.02 | 0.05 | -0.06 | -0.02 | 0.04 | -0.03 | -0.41 | 0.67 | 0.06 | 0.02 | -0.02 | 0.05 | 0.01 | -0.13 | 0.00 | -0.05 | -0.02 | -0.07 | -0.10 |
| Zhenglan | 0.07 | -0.05 | 0.01 | -0.10 | 0.03 | -0.01 | -0.51 | -0.18 | -0.07 | 0.03 | 0.06 | -0.01 | 0.47 | 0.26 | 0.04 | -0.06 | -0.10 | 0.02 | 0.27 | 0.10 | 0.03 | 0.04 | 0.02 | -0.02 |
| Zhengxiangbai | -0.30 | -0.11 | -0.04 | 0.08 | -0.13 | 0.00 | -0.34 | -0.10 | -0.12 | 0.03 | -0.08 | -0.17 | 0.64 | 0.18 | 0.03 | -0.01 | -0.04 | -0.03 | 0.26 | 0.14 | 0.03 | -0.14 | 0.12 | -0.05 |
| Zhungeer | 0.69 | 0.10 | 0.06 | 0.11 | 0.08 | -0.14 | 0.12 | 0.05 | 0.09 | -0.03 | -0.06 | -0.41 | -0.23 | 0.17 | 0.16 | 0.03 | 0.11 | -0.05 | 0.35 | -0.02 | -0.01 | 0.03 | 0.03 | -0.07 |
| Zhuozi | -0.11 | -0.02 | -0.01 | 0.01 | -0.02 | -0.16 | 0.38 | 0.09 | 0.10 | -0.07 | -0.02 | -0.24 | 0.19 | 0.22 | -0.02 | 0.04 | -0.12 | 0.12 | 0.36 | 0.11 | 0.01 | 0.01 | -0.24 | -0.07 |

Table S3. The information on dominant positive and negative drivers on four potential NCP in each county in Nei Mongol (values marked by yellow were used as examples in the section 3.4).

| County | Largest driver on HAB | Largest impact on HAB | Smallest driver on HAB | Smallest impact on HAB | Largest driver on NPP | Largest impact on NPP | Smallest driver on NPP | Smallest impact on NPP | Largest driver on PET | Largest impact on PET | Smallest driver on PET | Smallest impact on PET | Largest driver on SCC | Largest impact on SCC | Smallest driver on SCC | Smallest impact on SCC |
| --- | --- | --- | --- | --- | --- | --- | --- | --- | --- | --- | --- | --- | --- | --- | --- | --- |
| Abaga | VEG | 0.04 | PRE | -0.25 | POP | 0.05 | TEM | -0.23 | TEM | 0.45 | VEG | -0.07 | TEM | 0.45 | URB | -0.02 |
| Aershan | PRE | 0.16 | POP | -0.03 | PRE | 0.46 | URB | -0.05 | PRE | 0.76 | GDP | -0.02 | PRE | 0.26 | URB | -0.06 |
| Alashanyou | TEM | 0.05 | URB | -0.05 | PRE | 0.70 | GDP | -0.04 | VEG | 0.08 | PRE | -0.68 | GDP | 0.05 | URB | -0.46 |
| Alashanzuo | TEM | 0.31 | URB | -0.04 | PRE | 0.24 | POP | -0.06 | TEM | 0.53 | PRE | -0.12 | PRE | 0.15 | URB | -0.09 |
| Alukeerqin | VEG | 0.12 | PRE | -0.59 | PRE | 0.11 | VEG | -0.16 | PRE | 0.57 | GDP | -0.02 | TEM | 0.05 | PRE | -0.25 |
| Aohan | PRE | 0.18 | TEM | -0.20 | PRE | 0.03 | URB | -0.23 | PRE | 0.87 | GDP | -0.06 | GDP | 0.01 | TEM | -0.11 |
| Arong | PRE | 0.67 | POP | -0.05 | POP | 0.03 | PRE | -0.55 | TEM | 0.11 | PRE | -0.39 | POP | 0.01 | PRE | -0.47 |
| Baiyunebokuang | GDP | 0.47 | PRE | -0.20 | VEG | 0.52 | URB | -0.27 | PRE | 0.44 | GDP | -0.01 | GDP | 0.20 | TEM | -0.09 |
| Balinyou | VEG | 0.14 | URB | -0.13 | PRE | 0.24 | TEM | -0.06 | PRE | 0.42 | URB | -0.06 | PRE | 0.46 | POP | 0.00 |
| Balinzuo | PRE | 0.03 | URB | -0.25 | PRE | 0.19 | GDP | -0.08 | VEG | 0.18 | PRE | -0.38 | PRE | 0.38 | VEG | -0.23 |
| Chaharrightfrontbanner | VEG | 0.24 | GDP | -0.17 | VEG | 0.05 | URB | -0.28 | GDP | 0.07 | PRE | -0.37 | VEG | 0.13 | PRE | -0.23 |
| Chaharrightmiddlebanner | PRE | 0.64 | GDP | 0.01 | PRE | 0.41 | VEG | -0.12 | PRE | 0.63 | VEG | -0.06 | PRE | 0.44 | VEG | -0.16 |
| Chaharrightrearbanner | PRE | 0.10 | TEM | -0.33 | PRE | 0.02 | VEG | -0.29 | PRE | 0.33 | VEG | -0.30 | PRE | 0.35 | GDP | -0.05 |
| Chenbaerhu | TEM | 0.05 | PRE | -0.58 | PRE | 0.20 | TEM | -0.05 | PRE | 0.36 | TEM | -0.27 | PRE | 0.51 | TEM | -0.05 |
| Daerhanmaominganlianhe | PRE | 0.90 | TEM | -0.05 | PRE | 0.49 | VEG | -0.11 | PRE | 0.85 | POP | -0.01 | GDP | 0.26 | TEM | -0.24 |
| Dalate | PRE | 0.68 | URB | -0.26 | PRE | 0.18 | VEG | -0.35 | TEM | 0.14 | PRE | -0.21 | PRE | 0.30 | TEM | -0.18 |
| Dengkou | POP | 0.01 | URB | -0.09 | PRE | 0.35 | VEG | -0.33 | PRE | 0.36 | POP | 0.00 | GDP | 0.01 | PRE | -0.19 |
| Donghe | PRE | 0.25 | URB | -0.46 | PRE | 0.19 | URB | -0.28 | URB | 0.36 | PRE | -0.26 | TEM | 0.32 | URB | -0.29 |
| Dongsheng | PRE | 0.78 | VEG | -0.24 | PRE | 0.27 | URB | -0.13 | PRE | 0.32 | GDP | -0.42 | PRE | 0.21 | VEG | -0.26 |
| Dongwuzhumuqin | PRE | 0.12 | VEG | -0.14 | PRE | 0.40 | POP | -0.02 | URB | 0.06 | PRE | -0.64 | PRE | 0.26 | GDP | -0.04 |
| Duolun | POP | -0.05 | TEM | -0.19 | VEG | 0.13 | URB | -0.22 | VEG | 0.25 | TEM | -0.43 | PRE | 0.12 | POP | -0.01 |
| Eerguna | GDP | 0.13 | PRE | -0.46 | VEG | 0.05 | PRE | -0.18 | PRE | 0.25 | URB | -0.09 | PRE | 0.25 | POP | -0.16 |
| Ejina | PRE | 0.47 | TEM | -0.44 | URB | 0.12 | VEG | -0.30 | URB | 0.05 | PRE | -0.35 | PRE | 0.21 | TEM | -0.17 |
| Elunchun | PRE | 0.60 | GDP | 0.00 | PRE | 0.28 | URB | -0.14 | TEM | 0.43 | GDP | -0.03 | TEM | 0.22 | URB | -0.08 |
| Erlianhaote | PRE | 0.76 | POP | 0.02 | VEG | 0.15 | PRE | -0.10 | VEG | -0.01 | TEM | -0.55 | PRE | 0.08 | URB | -0.06 |
| Etuoke | PRE | 0.76 | URB | -0.06 | PRE | 0.67 | TEM | -0.19 | PRE | 0.50 | VEG | -0.05 | GDP | 0.07 | PRE | -0.12 |
| Etuokeqian | POP | 0.26 | TEM | -0.31 | URB | 0.06 | VEG | -0.35 | POP | 0.28 | URB | -0.25 | VEG | 0.19 | TEM | -0.15 |
| Ewenkezu | GDP | 0.02 | PRE | -0.83 | PRE | 0.18 | GDP | -0.09 | GDP | 0.06 | PRE | -0.07 | PRE | 0.57 | GDP | -0.06 |
| Fengzhen | PRE | 0.21 | GDP | -0.22 | VEG | 0.02 | GDP | -0.37 | URB | 0.12 | PRE | -0.78 | POP | 0.01 | VEG | -0.22 |
| Genhe | URB | 0.09 | PRE | -0.35 | POP | 0.06 | PRE | -0.48 | URB | -0.05 | PRE | -0.35 | PRE | 0.20 | URB | -0.10 |
| Guyang | PRE | 0.41 | VEG | -0.24 | PRE | 0.22 | VEG | -0.24 | PRE | 0.21 | VEG | -0.13 | PRE | 0.20 | VEG | -0.27 |
| Haibowan | PRE | 0.57 | GDP | -0.30 | URB | 0.34 | GDP | -0.19 | TEM | 0.16 | VEG | -0.01 | PRE | 0.21 | URB | -0.21 |
| Hailaer | VEG | 0.17 | PRE | -0.45 | TEM | 0.00 | URB | -0.36 | GDP | 0.15 | TEM | -0.19 | PRE | 0.40 | POP | -0.02 |
| Hainan | POP | -0.01 | TEM | -0.27 | PRE | 0.50 | GDP | -0.20 | GDP | 0.23 | URB | -0.18 | GDP | 0.06 | PRE | -0.30 |
| Hangjin | PRE | 0.85 | URB | -0.02 | PRE | 0.71 | TEM | -0.03 | VEG | 0.26 | GDP | -0.04 | PRE | 0.19 | POP | -0.07 |
| Hangjinhou | PRE | 0.18 | VEG | -0.19 | TEM | 0.05 | VEG | -0.43 | PRE | 0.42 | TEM | -0.18 | VEG | 0.20 | PRE | -0.09 |
| Helingeer | VEG | 0.34 | URB | -0.09 | PRE | 0.27 | VEG | -0.18 | GDP | 0.09 | VEG | -0.26 | VEG | 0.26 | URB | -0.08 |
| Hongshan | TEM | 0.03 | GDP | -0.69 | VEG | 0.22 | URB | -0.44 | PRE | 0.74 | GDP | -0.29 | PRE | 0.21 | VEG | -0.05 |
| Huade | PRE | 0.34 | VEG | -0.16 | GDP | 0.17 | URB | -0.23 | PRE | 0.56 | VEG | -0.03 | VEG | 0.16 | PRE | -0.06 |
| Huimin | VEG | 0.10 | URB | -0.68 | POP | 0.17 | URB | -0.76 | GDP | 0.35 | PRE | -0.72 | POP | 0.06 | GDP | -0.57 |
| Huolinguole | GDP | 0.22 | VEG | -0.35 | PRE | 0.17 | URB | -0.54 | PRE | 0.67 | VEG | 0.02 | PRE | 0.14 | URB | -0.22 |
| Jining | POP | 0.27 | URB | -0.36 | POP | 0.52 | VEG | -0.48 | GDP | 0.24 | PRE | -0.34 | URB | 0.18 | PRE | -0.17 |
| Jiuyuan | PRE | 0.56 | GDP | -0.28 | PRE | 0.22 | URB | -0.31 | GDP | 0.20 | PRE | -0.58 | PRE | 0.50 | GDP | -0.27 |
| Kailu | PRE | 0.55 | GDP | -0.04 | VEG | 0.07 | URB | -0.09 | TEM | 0.65 | PRE | -0.20 | GDP | 0.08 | PRE | -0.23 |
| Kalaqin | POP | -0.01 | PRE | -0.18 | GDP | 0.04 | URB | -0.46 | PRE | 0.88 | VEG | -0.09 | PRE | 0.59 | GDP | -0.01 |
| Kangbashi | PRE | 0.17 | GDP | -0.43 | GDP | 0.36 | URB | -0.30 | PRE | 0.34 | GDP | -0.02 | TEM | 0.10 | PRE | -0.15 |
| Keerqin | PRE | 0.25 | TEM | -0.13 | PRE | 0.25 | URB | -0.16 | PRE | 0.52 | URB | -0.02 | VEG | 0.03 | TEM | -0.44 |
| Keerqinyouyiqian | PRE | 0.51 | GDP | -0.13 | PRE | 0.67 | URB | -0.14 | PRE | 0.67 | VEG | -0.09 | VEG | 0.15 | PRE | -0.32 |
| Keerqinyouyizhong | VEG | 0.08 | PRE | -0.45 | VEG | 0.00 | URB | -0.10 | PRE | 0.83 | POP | -0.03 | VEG | 0.07 | PRE | -0.45 |
| Keerqinzuoyihou | GDP | 0.01 | URB | -0.13 | TEM | 0.25 | PRE | -0.07 | PRE | 0.68 | GDP | -0.03 | GDP | 0.08 | TEM | -0.30 |
| Keerqinzuoyizhong | PRE | 0.35 | TEM | -0.46 | PRE | 0.25 | URB | -0.12 | PRE | 0.72 | VEG | -0.07 | VEG | 0.12 | PRE | -0.63 |
| Keshiketeng | PRE | 0.24 | GDP | -0.04 | PRE | 0.11 | URB | -0.13 | PRE | 0.35 | GDP | -0.03 | TEM | 0.13 | PRE | -0.15 |
| Kulun | VEG | -0.01 | TEM | -0.26 | VEG | 0.37 | TEM | -0.11 | PRE | 0.32 | VEG | -0.04 | PRE | 0.03 | TEM | -0.14 |
| Kundulun | PRE | 0.25 | GDP | -0.45 | PRE | 0.17 | URB | -0.26 | VEG | 0.18 | PRE | -0.36 | PRE | 0.24 | GDP | -0.34 |
| Liangcheng | VEG | 0.25 | URB | -0.14 | PRE | 0.52 | URB | -0.17 | URB | 0.03 | PRE | -0.65 | PRE | 0.49 | VEG | -0.15 |
| Linhe | URB | 0.14 | PRE | -0.12 | GDP | 0.13 | VEG | -0.30 | URB | 0.15 | POP | 0.00 | PRE | 0.11 | TEM | -0.04 |
| Linxi | PRE | 0.05 | TEM | -0.21 | PRE | 0.11 | URB | -0.22 | TEM | 0.06 | VEG | -0.27 | PRE | 0.40 | URB | 0.02 |
| Manzhouli | GDP | 0.24 | TEM | -0.11 | VEG | 0.11 | URB | -0.19 | TEM | 0.59 | GDP | -0.02 | PRE | 0.06 | TEM | -0.13 |
| Molidawadawoerzu | PRE | 0.33 | TEM | -0.16 | PRE | 0.13 | URB | -0.15 | PRE | 0.32 | TEM | -0.19 | TEM | 0.28 | URB | -0.11 |
| Naiman | PRE | 0.25 | TEM | -0.49 | PRE | 0.26 | VEG | -0.06 | PRE | 0.19 | POP | 0.00 | VEG | 0.06 | TEM | -0.20 |
| Ningcheng | TEM | 0.00 | VEG | -0.07 | VEG | -0.02 | PRE | -0.51 | PRE | 0.85 | POP | -0.01 | URB | 0.20 | POP | 0.02 |
| Qingshan | PRE | 0.07 | GDP | -0.71 | POP | 0.05 | GDP | -0.46 | POP | 0.14 | PRE | -0.50 | PRE | 0.04 | GDP | -0.37 |
| Qingshuihe | PRE | 0.37 | URB | -0.05 | VEG | 0.06 | URB | -0.26 | PRE | 0.20 | VEG | -0.08 | PRE | 0.12 | URB | -0.09 |
| Saihan | PRE | 0.42 | GDP | -0.13 | PRE | 0.25 | URB | -0.37 | GDP | 0.30 | PRE | -0.42 | TEM | 0.32 | URB | -0.08 |
| Shangdu | VEG | 0.14 | GDP | -0.10 | POP | -0.01 | PRE | -0.24 | PRE | 0.45 | VEG | -0.18 | GDP | 0.03 | VEG | -0.06 |
| Shiguai | PRE | 0.01 | GDP | -0.35 | PRE | 0.31 | GDP | -0.18 | GDP | 0.23 | PRE | -0.40 | PRE | 0.24 | VEG | -0.15 |
| Siziwang | PRE | 0.77 | POP | -0.05 | PRE | 0.64 | VEG | -0.05 | PRE | 0.75 | POP | -0.07 | URB | 0.16 | VEG | -0.06 |
| Songshan | VEG | 0.07 | PRE | -0.56 | VEG | 0.07 | PRE | -0.23 | PRE | 0.93 | GDP | -0.01 | PRE | 0.40 | POP | -0.02 |
| Suniteyou | VEG | 0.09 | TEM | -0.15 | PRE | 0.09 | TEM | -0.22 | PRE | 0.19 | TEM | -0.28 | VEG | -0.03 | URB | -0.40 |
| Sunitezuo | PRE | 0.77 | URB | -0.09 | PRE | 0.30 | TEM | -0.27 | TEM | 0.32 | URB | -0.03 | TEM | 0.26 | PRE | -0.25 |
| Taipusi | PRE | 0.34 | URB | -0.09 | POP | 0.15 | URB | -0.11 | URB | 0.01 | PRE | -0.41 | VEG | 0.07 | PRE | -0.36 |
| Tumoteyou | PRE | 0.57 | URB | -0.15 | PRE | 0.26 | URB | -0.21 | GDP | 0.32 | PRE | -0.11 | PRE | 0.46 | URB | -0.12 |
| Tumotezuo | PRE | 0.71 | URB | -0.15 | PRE | 0.28 | URB | -0.33 | GDP | 0.10 | PRE | -0.55 | PRE | 0.56 | URB | -0.13 |
| Tuoketuo | PRE | 0.18 | POP | -0.09 | TEM | 0.05 | URB | -0.33 | TEM | 0.25 | GDP | -0.19 | TEM | 0.18 | URB | -0.03 |
| Tuquan | VEG | 0.38 | GDP | -0.14 | VEG | 0.14 | URB | -0.24 | PRE | 0.23 | VEG | -0.40 | VEG | 0.17 | PRE | -0.44 |
| Wengniute | VEG | 0.01 | PRE | -0.64 | POP | 0.02 | PRE | -0.28 | PRE | 0.87 | URB | -0.03 | TEM | 0.04 | PRE | -0.22 |
| Wuchuan | PRE | 0.20 | VEG | -0.21 | POP | 0.05 | VEG | -0.45 | GDP | 0.06 | PRE | -0.19 | URB | 0.04 | VEG | -0.23 |
| Wuda | GDP | 0.23 | TEM | -0.37 | TEM | 0.23 | VEG | -0.14 | TEM | 0.38 | GDP | -0.16 | GDP | 0.16 | PRE | -0.26 |
| Wulanhaote | PRE | 0.41 | URB | -0.06 | PRE | 0.42 | URB | -0.25 | VEG | 0.18 | PRE | -0.14 | GDP | 0.04 | VEG | -0.08 |
| Wulatehou | PRE | 0.52 | TEM | -0.08 | PRE | 0.32 | POP | -0.42 | TEM | 0.40 | VEG | -0.11 | PRE | 0.37 | TEM | -0.20 |
| Wulateqian | PRE | 0.55 | POP | -0.02 | PRE | 0.24 | VEG | -0.24 | GDP | 0.02 | PRE | -0.68 | PRE | 0.27 | TEM | -0.07 |
| Wulatezhong | PRE | 0.73 | POP | -0.04 | PRE | 0.60 | VEG | -0.20 | PRE | 0.54 | VEG | -0.16 | TEM | 0.10 | VEG | -0.06 |
| Wushen | PRE | 0.71 | TEM | -0.28 | PRE | 0.19 | VEG | -0.33 | TEM | 0.98 | PRE | -0.14 | VEG | 0.19 | TEM | -0.09 |
| Wuyuan | URB | 0.16 | GDP | -0.18 | GDP | 0.08 | URB | -0.10 | PRE | 0.77 | POP | 0.01 | VEG | 0.07 | PRE | -0.04 |
| Xianghuang | VEG | 0.16 | PRE | -0.36 | POP | 0.01 | PRE | -0.47 | PRE | 0.40 | VEG | -0.11 | PRE | 0.28 | VEG | -0.10 |
| Xilinhaote | PRE | 0.12 | TEM | -0.21 | POP | 0.06 | TEM | -0.33 | TEM | 0.38 | PRE | -0.41 | TEM | 0.13 | PRE | -0.29 |
| Xinbaerhuyou | PRE | 0.18 | URB | -0.10 | PRE | 0.24 | TEM | -0.19 | TEM | 0.26 | PRE | -0.44 | PRE | 0.22 | URB | -0.06 |
| Xinbaerhuzuo | POP | 0.02 | PRE | -0.53 | TEM | 0.05 | PRE | -0.19 | URB | 0.02 | PRE | -0.74 | PRE | 0.68 | POP | -0.01 |
| Xincheng | PRE | 0.20 | VEG | -0.44 | PRE | 0.28 | URB | -0.54 | GDP | 0.24 | PRE | -0.81 | TEM | 0.07 | VEG | -0.31 |
| Xinghe | VEG | 0.09 | PRE | -0.26 | POP | 0.03 | PRE | -0.46 | VEG | 0.06 | PRE | -0.77 | POP | 0.01 | PRE | -0.37 |
| Xiwuzhumuqin | PRE | 0.39 | TEM | -0.09 | PRE | 0.21 | URB | -0.36 | GDP | 0.02 | PRE | -0.41 | PRE | 0.46 | TEM | -0.05 |
| Yakeshi | PRE | 0.23 | VEG | -0.12 | PRE | 0.34 | URB | -0.08 | PRE | 0.71 | URB | -0.03 | PRE | 0.45 | GDP | -0.13 |
| Yijinhuoluo | PRE | 0.63 | VEG | -0.08 | PRE | 0.31 | VEG | -0.35 | PRE | 0.50 | VEG | -0.06 | PRE | 0.25 | POP | -0.08 |
| Yuanbaoshan | GDP | 0.04 | TEM | -0.37 | GDP | 0.01 | URB | -0.41 | TEM | 0.32 | VEG | -0.27 | PRE | 0.19 | GDP | -0.09 |
| Yuquan | POP | 0.23 | GDP | -0.24 | PRE | 0.14 | GDP | -0.39 | GDP | 0.02 | PRE | -0.37 | URB | 0.34 | GDP | -0.09 |
| Zhalainuoer | GDP | 0.38 | POP | -0.04 | POP | 0.18 | URB | -0.54 | TEM | 0.27 | GDP | -0.33 | POP | 0.02 | TEM | -0.31 |
| Zhalaite | POP | 0.00 | PRE | -0.12 | VEG | 0.08 | TEM | -0.14 | PRE | 0.64 | VEG | -0.03 | GDP | 0.00 | PRE | -0.47 |
| Zhalantun | PRE | 0.32 | GDP | -0.06 | PRE | 0.00 | GDP | -0.08 | POP | 0.00 | PRE | -0.80 | POP | 0.04 | URB | -0.14 |
| Zhalute | TEM | 0.09 | PRE | -0.28 | PRE | 0.05 | URB | -0.41 | PRE | 0.67 | GDP | -0.02 | TEM | 0.00 | PRE | -0.13 |
| Zhenglan | PRE | 0.07 | GDP | -0.10 | VEG | 0.06 | PRE | -0.51 | PRE | 0.47 | VEG | -0.10 | PRE | 0.27 | URB | -0.02 |
| Zhengxiangbai | GDP | 0.08 | PRE | -0.30 | GDP | 0.03 | PRE | -0.34 | PRE | 0.64 | VEG | -0.04 | PRE | 0.26 | GDP | -0.14 |
| Zhungeer | PRE | 0.69 | URB | -0.14 | PRE | 0.12 | URB | -0.41 | TEM | 0.17 | PRE | -0.23 | PRE | 0.35 | URB | -0.07 |
| Zhuozi | GDP | 0.01 | URB | -0.16 | PRE | 0.38 | URB | -0.24 | TEM | 0.22 | VEG | -0.12 | PRE | 0.36 | VEG | -0.24 |

Table S4. The information on quantitative pairwise relationships of four potential NCP in each county in Nei Mongol (values marked by yellow were used as examples in the section 3.5; values marked by blue were used as examples in the section 4.1).

| County | HAB<->NPP | HAB<->PET | HAB<->SCC | NPP<->PET | NPP<->SCC | PET<->SCC |
| --- | --- | --- | --- | --- | --- | --- |
| Abaga | -0.0092 | -0.0934 | 0.0715 | -0.0726 | -0.1458 | 0.2492 |
| Aershan | -0.2327 | 0.1972 | -0.2375 | -0.0538 | 0.1423 | 0.0097 |
| Alashanyou | 0.0136 | -0.0183 | 0.1671 | 0.2293 | 0.0090 | 0.0468 |
| Alashanzuo | 0.3485 | -0.2218 | 0.1721 | -0.3332 | 0.1277 | 0.0636 |
| Alukeerqin | 0.0041 | -0.2918 | 0.4027 | 0.1756 | 0.0846 | -0.2102 |
| Aohan | 0.2336 | -0.2504 | 0.1589 | -0.1251 | 0.1006 | -0.2073 |
| Arong | -0.2911 | 0.1460 | -0.2641 | 0.0461 | 0.2616 | -0.0588 |
| Baiyunebokuang | 0.0741 | -0.3050 | 0.2386 | -0.0556 | -0.1400 | 0.0273 |
| Balinyou | -0.1960 | 0.2517 | 0.0722 | -0.0271 | 0.1089 | 0.1274 |
| Balinzuo | -0.0615 | -0.1313 | 0.3236 | 0.1255 | 0.0887 | -0.2305 |
| Chaharrightfrontbanner | 0.3617 | 0.0374 | 0.4766 | -0.1427 | 0.2124 | 0.2455 |
| Chaharrightmiddlebanner | 0.5866 | -0.1970 | 0.3879 | -0.1950 | 0.4317 | 0.1228 |
| Chaharrightrearbanner | 0.4221 | 0.0309 | -0.0815 | 0.2676 | 0.0933 | -0.0899 |
| Chenbaerhu | 0.1337 | 0.3448 | -0.3263 | 0.1431 | 0.0434 | -0.0471 |
| Daerhanmaominganlianhe | 0.0547 | 0.4382 | 0.2152 | 0.0826 | -0.1133 | 0.0552 |
| Dalate | -0.2721 | -0.7002 | 0.4508 | 0.3889 | -0.2275 | -0.2818 |
| Dengkou | -0.4093 | -0.4884 | 0.6706 | 0.3251 | -0.2761 | -0.4196 |
| Donghe | -0.0019 | -0.4812 | 0.5667 | -0.0410 | 0.0167 | -0.2566 |
| Dongsheng | -0.0362 | 0.4477 | 0.3347 | -0.2565 | -0.0433 | 0.2509 |
| Dongwuzhumuqin | 0.1758 | -0.0926 | 0.3086 | -0.1666 | 0.0774 | 0.0323 |
| Duolun | 0.2880 | -0.1504 | -0.4946 | 0.1366 | -0.2078 | -0.0592 |
| Eerguna | -0.1940 | -0.7698 | -0.2996 | 0.0823 | 0.0160 | 0.3496 |
| Ejina | -0.2155 | 0.1313 | 0.1902 | -0.0068 | -0.0125 | 0.1890 |
| Elunchun | -0.1460 | 0.0299 | -0.1900 | 0.0278 | 0.2482 | -0.0641 |
| Erlianhaote | 0.0229 | -0.3637 | 0.1107 | 0.1323 | 0.0698 | 0.0346 |
| Etuoke | -0.0355 | -0.0599 | 0.4617 | -0.1316 | -0.0733 | 0.0265 |
| Etuokeqian | 0.2438 | 0.6946 | 0.3577 | 0.1999 | 0.1154 | 0.1315 |
| Ewenkezu | -0.0829 | 0.1745 | -0.0852 | -0.0859 | -0.1185 | 0.0289 |
| Fengzhen | 0.1521 | -0.2882 | 0.4769 | -0.0934 | 0.0815 | -0.0355 |
| Genhe | 0.1900 | -0.4420 | -0.0534 | -0.1527 | 0.1105 | 0.0302 |
| Guyang | 0.2168 | 0.1901 | 0.5648 | -0.0472 | 0.3303 | 0.1105 |
| Haibowan | 0.2567 | -0.0728 | 0.4832 | -0.0310 | 0.1493 | -0.0971 |
| Hailaer | 0.0135 | -0.0105 | 0.0564 | 0.0436 | 0.0231 | 0.0265 |
| Hainan | -0.0634 | 0.1188 | 0.4977 | -0.2150 | -0.0538 | 0.0377 |
| Hangjin | 0.2506 | -0.3242 | 0.1489 | 0.0276 | -0.0042 | 0.0838 |
| Hangjinhou | -0.0186 | 0.2605 | -0.0977 | 0.0773 | -0.1002 | -0.2011 |
| Helingeer | -0.0158 | -0.1649 | 0.2872 | -0.3426 | 0.0171 | -0.1432 |
| Hongshan | 0.2052 | 0.0337 | 0.1005 | -0.0643 | 0.0694 | 0.0185 |
| Huade | 0.0396 | 0.3345 | -0.4672 | 0.3378 | 0.0373 | 0.0310 |
| Huimin | 0.0476 | -0.0434 | 0.0450 | 0.0119 | 0.3268 | -0.2762 |
| Huolinguole | 0.1440 | 0.0525 | 0.1018 | -0.0633 | 0.2464 | -0.2303 |
| Jining | 0.6217 | 0.4140 | -0.2375 | 0.2767 | -0.0583 | 0.0939 |
| Jiuyuan | 0.2383 | -0.1930 | 0.6992 | -0.0534 | 0.3273 | -0.1904 |
| Kailu | -0.0029 | 0.0718 | 0.0984 | 0.0804 | -0.0197 | 0.0295 |
| Kalaqin | 0.0426 | -0.0319 | 0.3882 | -0.0554 | -0.0674 | -0.0727 |
| Kangbashi | -0.0191 | -0.0771 | 0.2452 | -0.1047 | -0.0128 | 0.0120 |
| Keerqin | -0.0480 | -0.0622 | 0.0358 | -0.0511 | -0.0019 | -0.2869 |
| Keerqinyouyiqian | 0.1008 | 0.2719 | -0.3181 | 0.0152 | 0.0255 | -0.1779 |
| Keerqinyouyizhong | 0.3389 | -0.5360 | 0.1918 | -0.4089 | 0.2497 | -0.2700 |
| Keerqinzuoyihou | 0.0050 | 0.0737 | 0.1454 | -0.1015 | -0.0699 | 0.2366 |
| Keerqinzuoyizhong | 0.0339 | 0.2877 | -0.0692 | 0.1510 | -0.0456 | -0.1681 |
| Keshiketeng | 0.2427 | -0.2732 | -0.2697 | -0.2568 | -0.0757 | 0.0678 |
| Kulun | -0.0338 | -0.3164 | 0.1175 | 0.0248 | 0.0713 | 0.1235 |
| Kundulun | 0.2491 | 0.3697 | 0.3546 | 0.2452 | 0.2518 | 0.0368 |
| Liangcheng | 0.2145 | 0.1393 | 0.3149 | 0.2054 | 0.1494 | -0.0438 |
| Linhe | -0.0401 | 0.2299 | 0.0501 | -0.1400 | -0.0512 | 0.1120 |
| Linxi | 0.0705 | -0.0105 | -0.3183 | 0.0768 | 0.1627 | 0.2909 |
| Manzhouli | -0.2918 | -0.3157 | -0.3719 | -0.0126 | 0.1826 | 0.1571 |
| Molidawadawoerzu | -0.2547 | 0.1031 | -0.3419 | -0.0172 | 0.1227 | -0.1808 |
| Naiman | 0.0200 | 0.1385 | 0.1922 | 0.1520 | 0.0017 | -0.0696 |
| Ningcheng | 0.0581 | 0.2943 | 0.0160 | -0.2974 | -0.2191 | 0.1873 |
| Qingshan | 0.5741 | -0.2653 | 0.4237 | -0.3092 | 0.4996 | -0.1547 |
| Qingshuihe | -0.3954 | 0.5854 | 0.0617 | -0.4687 | 0.0400 | -0.0093 |
| Saihan | 0.3685 | -0.5332 | 0.6174 | -0.1781 | 0.3484 | -0.2692 |
| Shangdu | -0.0420 | -0.1892 | -0.5307 | 0.2964 | 0.0969 | 0.3589 |
| Shiguai | 0.1580 | -0.1341 | 0.1246 | -0.3666 | 0.2575 | -0.0982 |
| Siziwang | 0.0443 | 0.0713 | 0.1864 | 0.4152 | 0.0127 | -0.0267 |
| Songshan | 0.1998 | -0.4039 | 0.0052 | -0.1720 | 0.0050 | 0.0523 |
| Suniteyou | 0.0095 | -0.0193 | 0.0050 | 0.3650 | 0.0583 | 0.1056 |
| Sunitezuo | 0.0784 | 0.3474 | 0.1273 | -0.0983 | -0.0781 | 0.1806 |
| Taipusi | 0.0870 | -0.1446 | -0.4507 | -0.2391 | -0.1152 | 0.2191 |
| Tumoteyou | 0.0703 | -0.2528 | 0.4771 | -0.1496 | 0.2269 | -0.1934 |
| Tumotezuo | 0.2957 | 0.1181 | 0.5717 | -0.0225 | 0.3656 | -0.0660 |
| Tuoketuo | -0.0761 | 0.1753 | 0.3743 | 0.1183 | -0.0599 | 0.0029 |
| Tuquan | 0.3784 | -0.6297 | 0.2899 | -0.2027 | 0.1033 | -0.4198 |
| Wengniute | 0.1806 | -0.2432 | 0.2826 | -0.1158 | 0.1191 | 0.0874 |
| Wuchuan | 0.4748 | 0.0304 | 0.5731 | 0.0619 | 0.3848 | 0.1828 |
| Wuda | -0.2241 | -0.4679 | 0.4225 | 0.3584 | -0.1659 | -0.2399 |
| Wulanhaote | 0.1805 | -0.5504 | 0.4955 | 0.0260 | 0.0997 | -0.4589 |
| Wulatehou | 0.3154 | -0.2092 | 0.4537 | 0.0256 | 0.3523 | -0.0051 |
| Wulateqian | -0.1670 | 0.0921 | 0.5881 | 0.1146 | -0.0525 | -0.0077 |
| Wulatezhong | 0.2307 | 0.1319 | 0.3835 | 0.2789 | 0.0177 | 0.0695 |
| Wushen | 0.1853 | -0.5909 | -0.0130 | -0.2146 | -0.0937 | 0.0616 |
| Wuyuan | -0.1649 | 0.1061 | 0.0559 | -0.0280 | 0.0729 | -0.1066 |
| Xianghuang | 0.3844 | 0.1228 | -0.4692 | 0.2789 | -0.1669 | -0.0700 |
| Xilinhaote | 0.1031 | -0.2090 | 0.1052 | -0.1068 | -0.1512 | 0.3197 |
| Xinbaerhuyou | 0.0408 | 0.0544 | -0.1256 | 0.0090 | 0.1258 | 0.1212 |
| Xinbaerhuzuo | -0.1039 | -0.3800 | -0.3217 | 0.1926 | 0.0318 | 0.0821 |
| Xincheng | 0.4460 | 0.0802 | 0.4071 | 0.0217 | 0.2219 | 0.2915 |
| Xinghe | 0.0852 | 0.5388 | 0.4760 | 0.2891 | 0.1928 | 0.3985 |
| Xiwuzhumuqin | 0.0715 | -0.0027 | 0.2011 | 0.2026 | 0.1300 | 0.1476 |
| Yakeshi | -0.1483 | 0.1231 | -0.2012 | 0.2291 | 0.2735 | 0.1421 |
| Yijinhuoluo | -0.0087 | 0.2411 | 0.3494 | -0.0057 | 0.0131 | 0.1172 |
| Yuanbaoshan | 0.2440 | -0.3717 | 0.2607 | -0.2513 | 0.0449 | -0.1811 |
| Yuquan | -0.0366 | 0.2627 | -0.1432 | -0.1754 | -0.0706 | -0.1641 |
| Zhalainuoer | -0.4301 | 0.3011 | -0.5134 | 0.0230 | 0.2936 | -0.0986 |
| Zhalaite | 0.1938 | -0.6006 | -0.0249 | -0.3452 | 0.2611 | -0.2245 |
| Zhalantun | -0.1731 | 0.2185 | -0.4234 | 0.2002 | 0.1936 | 0.0020 |
| Zhalute | 0.1855 | -0.6154 | 0.4331 | -0.1915 | 0.2767 | -0.3030 |
| Zhenglan | -0.0001 | -0.2319 | -0.4525 | -0.1797 | -0.0884 | 0.0366 |
| Zhengxiangbai | 0.3294 | -0.3634 | -0.5506 | -0.2449 | -0.2006 | 0.2606 |
| Zhungeer | 0.1680 | 0.1066 | 0.3242 | 0.0907 | 0.0440 | 0.1057 |
| Zhuozi | 0.1554 | 0.1061 | 0.1538 | -0.0630 | 0.2743 | 0.2334 |
